# The Role of Local Triplet Excited States and D‐A Relative Orientation in Thermally Activated Delayed Fluorescence: Photophysics and Devices

**DOI:** 10.1002/advs.201600080

**Published:** 2016-07-18

**Authors:** Fernando B. Dias, Jose Santos, David R. Graves, Przemyslaw Data, Roberto S. Nobuyasu, Mark A. Fox, Andrei S. Batsanov, Tiago Palmeira, Mário N. Berberan‐Santos, Martin R. Bryce, Andrew P. Monkman

**Affiliations:** ^1^ Physics DepartmentDurham UniversitySouth RoadDurhamDH1 3LEUK; ^2^ IMDEA NanocienciaC/Faraday, 9Campus Universitario de Cantoblanco28049MadridSpain; ^3^ Faculty of ChemistrySilesian University of TechnologyM. Strzody 944‐100GliwicePoland; ^4^ Chemistry DepartmentDurham UniversitySouth RoadDurhamDH1 3LEUK; ^5^ Centro de Quimica‐Fisica MolecularInstituto Superior Tecnico1049‐001LisboaPortugal

**Keywords:** charge transfer, electroluminescence, fluorescence, OLEDs, TADF

## Abstract

Here, a comprehensive photophysical investigation of a the emitter molecule **DPTZ‐DBTO2**, showing thermally activated delayed fluorescence (TADF), with near‐orthogonal electron donor (D) and acceptor (A) units is reported. It is shown that **DPTZ‐DBTO2** has minimal singlet–triplet energy splitting due to its near‐rigid molecular geometry. However, the electronic coupling between the local triplet (^3^LE) and the charge transfer states, singlet and triplet, (^1^CT, ^3^CT), and the effect of dynamic rocking of the D–A units about the orthogonal geometry are crucial for efficient TADF to be achieved. In solvents with low polarity, the guest emissive singlet ^1^CT state couples directly to the near‐degenerate ^3^LE, efficiently harvesting the triplet states by a spin orbit coupling charge transfer mechanism (SOCT). However, in solvents with higher polarity the emissive CT state in **DPTZ‐DBTO2** shifts below (the static) ^3^LE, leading to decreased TADF efficiencies. The relatively large energy difference between the ^1^CT and ^3^LE states and the extremely low efficiency of the ^1^CT to ^3^CT hyperfine coupling is responsible for the reduction in TADF efficiency. Both the electronic coupling between ^1^CT and ^3^LE, and the (dynamic) orientation of the D–A units are thus critical elements that dictate reverse intersystem crossing processes and thus high efficiency in TADF.

## 1 Introduction

External quantum efficiencies (EQEs) as large as 25% have been reported recently in organic light emitting diodes (OLEDs) using intramolecular charge transfer emitters which are capable of harvesting nonemissive triplet states and converting them to singlet excited states via thermally activated reverse intersystem crossing (RISC).^1^ Triplet state harvesting via this mechanism leads to thermally activated delayed fluorescence (TADF) or E‐type delayed fluorescence, and overcomes the spin statistical 25% limit on the singlet production yield arising from direct charge recombination.^2^ The efficiency of the RISC mechanism, and therefore the contribution of TADF to the overall emission, increases when two conditions are fulfilled: (i) the nonradiative internal conversion pathways, available for the excited singlet (S_1_) and triplet (T_1_) states, are suppressed and (ii) a small energy splitting between the lowest singlet and triplet states is achieved.^3^ These two conditions require molecular structures with strong intramolecular charge transfer (CT) character, where excited state molecular conformations break the conjugation between the donor (D) and acceptor (A) units.^4^

**Figure 1:**
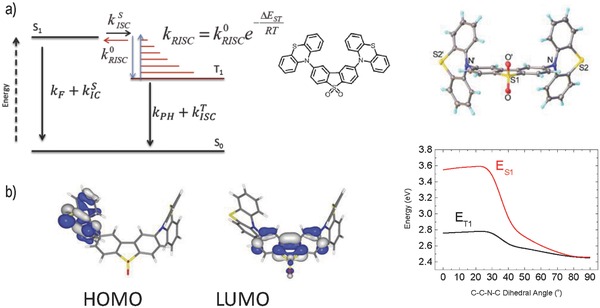
a) Simplified energy diagram showing the thermally assisted equilibrium between singlet and triplet excited states; chemical structure and X‐ray molecular structure of **DPTZ‐DBTO2**,^.^2CDCl_3_, CCDC‐1034115. b) HOMO and LUMO of **DPTZ‐DBTO2**, and S_1_ and T_1_ energies obtained from TD‐DFT calculations at B3LYP/6‐31G(d), as a function of the D–A–D relative dihedral angles, assuming that singlet and triplet excited state geometries are the same.

Several CT molecules have been reported with triplet‐harvesting efficiencies close to 100%,^2^, ^5^ leading to significantly increased device EQEs. However, while great progress has been made on improving device efficiencies, the way molecular structure and the ordering of energy levels affect the efficiency of TADF, still remains unclear, as does the RISC mechanism itself as we shall show here. From the rigorous photophysical investigation of the D–A–D molecule **DPTZ‐DBTO2** (**Figure** **1**a) we elucidate the role of local triplet states and CT states, and the role of the D to A orientation in the TADF mechanism. **DPTZ‐DBTO2** comprises two phenothiazine electron donors covalently linked to a central dibenzothiophene‐*S,S*‐dioxide acceptor, and follows on from the family of D–A–D molecules we have previously reported.[[qv: 2b,6]] The previous molecules do not have constrained structures like **DPTZ‐DBTO2** thus leading to large heterogeneities in photophysical and device properties.

In light of our recently reported results with D–A and D–A–D analogues of **DPTZ‐DBTO2**,^7^, ^8^ here we show that while a rigid near‐orthogonal D–A–D molecular geometry is crucial to minimize the singlet–triplet energy gap, the efficiency of TADF is ultimately controlled by two other factors: i) the electronic coupling between the local triplet, (^3^LE), and the charge transfer singlet states, (^1^CT, ^3^CT) yielding interconversion of singlet and triplet states by the spin orbit charge transfer mechanism, a second order process mediated by vibronic coupling,^9^ and ii) the need for the D–A geometry to be dynamically rocking about D–A orthogonality. When the molecule is rigidly constrained in the orthogonal D–A geometry no TADF is observed,^8^ since electron‐coupling between ^1^LE and ^1^CT is so weak that it is out competed by intersystem crossing (ISC) and internal conversion (IC) and no electron transfer is able to occur (also the ^1^CT cannot couple radiatively to the ground state), but the singlet–triplet gap is minimized. In contrast, if the D–A geometry is allowed to relax toward planarity, the singlet–triplet energy gap rapidly opens up preventing RISC, and TADF becomes inefficient. Therefore, an optimum D–A geometry exists that maximizes TADF. Moreover, as we previously observed^7^ and here analyse in full detail, the electronic coupling between the ^1^CT singlet and the local triplet ^3^LE strongly influences the TADF efficiency. Shifting the ^1^CT state away from the local triplet state ^3^LE leads to a significant quenching of the TADF emission.

During the preparation of this manuscript Tang and co‐workers^10^ published an independent synthesis of **DPTZ‐DBTO2**, focusing on the observation of aggregation induced emission and delayed fluorescence. However, this article did not show photophysical evidence of TADF in **DPTZ‐DBTO2**, or device data, which we report here in detail to reveal new insights into the TADF mechanism.

## 2 Results and Discussion

### 2.1 X‐Ray, DFT, and CV Data

The conformation of the D and A subunits in **DPTZ‐DBTO2** yields very small overlap between the Highest Occupied Molecular Orbital (HOMO) and Lowest Unnocupied Molecular Orbital (LUMO) (Figure S2, Supporting Information), and gives rise to a singlet excited state with strong charge transfer character. This is further supported by the X‐ray molecular structure, which shows a configuration that minimizes the overlap of the phenothiazine N‐10 lone pairs with the dibenzothiophene‐*S,S*‐dioxide unit, Figure 1a. The **DPTZ‐DBTO2** molecule has crystallographic C_2_ symmetry, the phenothiazine units are folded by 34° along N…S vectors and on average are inclined by 82° to the planar dibenzothiophene‐*S,S*‐dioxide π‐system. The subunits in **DPTZ‐DBTO2** are thus sterically “locked” in nearly perpendicular D–A orientation, confirmed by single‐crystal X‐ray data, Figure 1a, (Figures S1 and S23, Supporting Information) and Time Dependent Density Functional Theory (TD‐DFT) calculations, Figure 1b. This conformation is shown to be dominant in controlling both the photophysics and device physics.

The HOMO and LUMO energy levels of **DPTZ‐DBTO2** were measured by cyclic voltammetry (CV), −5.2 ± 0.1 eV and −3.0 ± 0.1 eV, respectively, in excellent agreement with the corresponding individual D (HOMO: −5.2 ± 0.1 eV) and A (LUMO: −2.9 ± 0.1 eV) unit energies, (Figure S6, Supporting Information), showing that the HOMO–LUMO gap in **DPTZ‐DBTO2** is determined by the HOMO and LUMO levels of the donor and acceptor, respectively.

The energies of the lowest singlet and triplet excited states of **DPTZ‐DBTO2**, as a function of D–A dihedral angle were determined from TD‐DFT calculations, Figure 1b. The excited state molecular geometries were optimized in the ground states with both dihedral angles constrained. Importantly, the singlet and triplet energies are strongly dependent on the D–A orientation, with the Δ*E*_ST_ energy varying between 0.8 eV for a planar geometry, and less than 0.01 eV for a D–A dihedral angle larger than 80° (Figure 1b). The near‐orthogonality between the D–A units is thus key to achieving almost degenerate singlet and triplet levels.

### 2.2 Photophysics in Solution

The absorption spectrum of **DPTZ‐DBTO2** clearly reflects the sum of phenothiazine (D) and dibenzothiophenedioxide (A) contributions, indicating negligible conjugation across the donor–acceptor units, which are almost entirely electronically decoupled. Following optical excitation, mainly of the phenothiazine unit at 337 nm (3.68 eV), the **DPTZ‐DBTO2** emission in nonpolar methylcyclohexane (MCH) appears broad, peaking at around 540 nm (2.30 eV), in excellent agreement with the energy difference between the HOMO and LUMO levels of phenothiazine and dibenzothiophene‐*S,S*‐dioxide, respectively (2.2 ± 0.1 eV) determined by CV data. The emission is strongly redshifted relative to the D and A emissions, even in nonpolar solvent, and no ^1^LE (^1^D or ^1^A) emission is observed in solution at room temperature (RT). However, residual ^1^LE (^1^D and ^1^A) emissions are detected around 450 nm in zeonex.

**Figure 2:**
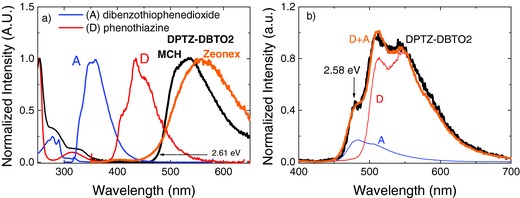
a) Absorption and emission spectra of **DPTZ‐DBTO2** in MCH, compared with spectra of the individual D and A fragments; comparison between the **DPTZ‐DBTO2** fluorescence in MCH and zeonex. b) **DPTZ‐DBTO2** phosphorescence spectra in zeonex at 80 K (black), compared with the phosphorescence of the individual D (red) and A (blue) fragments.

The emissive singlet state in **DPTZ‐DBTO2** is thereafter identified as the ^1^CT state, emitting at 2.61 ± 0.02 eV in MCH at RT, as determined from the emission onset, see **Figure** **2**a. With increasing polarity the **DPTZ‐DBTO2** emission redshifts, peaking at 580 nm (2.13 eV) in toluene, and at 605 nm (2.05 eV) in chlorobenzene, but the intensity decreases significantly (Figure S10, Supporting Information). Complete emission quenching is observed in strong polar media, such as ethanol, as is normally observed in exciplex and twisted intramolecular charge transfer states,^11^ giving clear indication that the emission originates from an excited state with strong charge transfer character.

**Figure 3:**
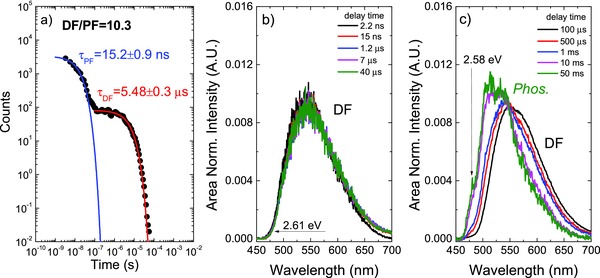
a) Plot of the **DPTZ‐DBTO2** emission intensity over a time interval spanning 8 orders of magnitude. b,c) **DPTZ‐DBTO2** time resolved area normalized emission spectra, obtained in MCH at 290 and 100 K, respectively.

The **DPTZ‐DBTO2** phosphorescence is excellently reproduced by the superposition of the phosphorescence emissions of the donor molecule phenothiazine (^3^D, 2.46 ± 0.02 eV) and the acceptor molecule dibenzothiophene‐*S,S*‐dioxide (^3^A, 2.62 ± 0.02 eV), with a shift of just 0.03 eV, showing that the lowest triplet state in **DPTZ‐DBTO2** is a local triplet state (^3^LE), which carries clear contributions from both the triplet of the donor, ^3^D, and the triplet of the acceptor, ^3^A. This is due to the near orthogonality between the D and A fragments in **DPTZ‐DBTO2** which decreases the electronic coupling between the two units. However, despite ^3^A and ^3^D having different phosphorescence lifetimes (130 and 64 μs, respectively), the ^3^LE phosphorescence of **DPTZ‐DBTO2** shows a single exponential decay without any change of the profile of the emission spectra over time (**Figure** **3**c). The phosphorescence of **DPTZ‐DBTO2** thus carries contributions from both ^3^A and ^3^D during the entire phosphorescence lifetime as a single ^3^LE triplet state, as seen in Figure 2b. The **DPTZ‐DBTO2** phosphorescence emitting from a local triplet excited state (^3^LE) is identified by its well‐resolved phosphorescence at low temperature in both solution and solid films, Figure 2b and Figure 3c, and the energy of the ^3^LE state is measured at the first vibronic peak as 2.58 eV.

Simultaneous emission from two triplet states has been reported before in other compounds, suggesting that Kasha's rule for internal conversion is not fully obeyed in these cases.^12^ However, the single exponential decay of **DPTZ‐DBTO2** phosphorescence is not compatible with two independent triplet states (^3^D and ^3^A) emitting simultaneously, and so here we observe phosphorescence from the unique lowest triplet excited state, which is of local character, (^3^LE). In zeonex the emission from ^1^CT appears slightly redshifted, due to the larger dielectric constant of zeonex, compared with MCH, a tail around 450 nm is also observed, due to a weak contribution from ^1^LE (^1^D and ^1^A) emissions. As is seen, in MCH the ^1^CT state is just 0.03 eV above ^3^LE.

Due to the vanishing Δ*E*_ST_ energy, a strong TADF contribution to the overall emission is observed in **DPTZ‐DBTO2**. For example, upon degassing in MCH, the luminescence of **DPTZ‐DBTO2**, increases ≈12 times (Figure S7, Supporting Information), and the fluorescence yield, *Φ*_F_, increases from 0.03 ± 0.01 to 0.3 ± 0.1. Oxygen very effectively quenches any triplet population but does not affect singlet states, thus in aerated solution only “prompt” ^1^CT emission is observed. The emission spectra obtained in aerated and degassed MCH solutions match each other exactly; showing that TADF (delayed fluorescence, DF) and prompt fluorescence (PF) come from the same ^1^CT state. The (dominant) TADF contribution to the overall **DPTZ‐DBTO2** emission is directly determined by comparing the emission in aerated and degassed solutions. In MCH, the recycled triplets^3^ contribute around 91% to the total **DPTZ‐DBTO2** fluorescence.

An excellent spectral match is also obtained between the steady state and delayed fluorescence (TADF) spectra of **DPTZ‐DBTO2** in MCH solution, again giving clear indication that both emissions come from the same excited state (^1^CT), (Figure S8, Supporting Information). The intramolecular origin of the delayed fluorescence (TADF) is confirmed by the strictly linear dependence of TADF with laser excitation intensity, and therefore, the origin of the delayed emission is unambiguously assigned to a thermally assisted mechanism, not triplet–triplet annihilation (Figure S9, Supporting Information). This is also true for **DPTZ‐DBTO2** in the solid state (Figure S17, Supporting Information). The **DPTZ‐DBTO2** luminescence decay, followed over a time interval spanning eight decades, shows two clear exponential components, Figure 3a: an initial decay term with a time constant of 15 ns, which is associated with the prompt ^1^CT fluorescence, and a second regime decaying with a 5.5 μs time constant, assigned to the TADF decay (DF). A 10.3 TADF/PF ratio (of the emission integrals) is determined from the time resolved decay. This is in excellent agreement with the TADF/PF ratio determined from steady‐state oxygen dependent experiments.

Time resolved, area normalized, emission spectra, obtained at 290 and 100 K, are shown in Figure 3b,c. At 290 K the emission is completely dominated by a single species, i.e., no isoemissive point is observed during the entire emission decay profile. However, at 100 K, below the freezing point of MCH, an isoemissive point is observed at late delay times, indicating two different excited state species; ^1^CT (prompt and delayed) fluorescence, and a long‐lived and resolved emission, observed only at low temperatures, assigned to the phosphorescence from the local triplet state (^3^LE).^13^

For strong TADF to occur, the following inequalities must hold (see Figure 1): $k_{\mathrm{ISC}}^{S}>>k_{F}+k_{\mathrm{IC}}^{S}$ and $k_{\mathrm{RISC}}>>k_{\mathrm{PH}}+k_{\mathrm{ISC}}^{T}$. In most cases, it is also observed that $k_{\mathrm{ISC}}^{S}>>k_{\mathrm{RISC}}$ and $k_{\mathrm{ISC}}^{T}>>k_{\mathrm{PH}}$. Interconversion of the singlet and triplet emissive states then occurs many times before photon emission or nonradiative decay can take place.^3^ Very strong TADF is clearly occurring in **DPTZ‐DBTO2** in MCH, and the yield of excited singlet states formed via reverse intersystem crossing, $\Phi_{S}^{T}$, can be assumed to be close to 1. The triplet yield, $\Phi_{T}$, is thus determined directly from the 10.3 DF/PF ratio as 91 ± 2%.[[qv: 2b,3]] Using the prompt luminescence lifetime, *τ*_F_ = 15.2 ± 0.9 ns, the intersystem crossing rate between the ^1^CT and local excited triplet states (^3^LE), $k_{\mathrm{ISC}}^{S}=\frac{\Phi_{T}}{\tau_{F}}=(5.9\;\pm \;0.3)\;\times \;10^{7}s^{-1}$ is also determined.

Using the fluorescence quantum yield (PLQY) of the prompt ^1^CT emission in **DPTZ‐DBTO2**, Φ_F_ = 0.03 ± 0.01, determined in MCH in the presence of oxygen, and the fluorescence lifetime, both the rate of natural radiative decay, $k_{F}=(0.19\;\pm \;0.01)\;\times \;10^{7}s^{-1}$, and the rate of internal conversion $k_{\mathrm{IC}}^{S}=(0.51\;\pm \;0.01)\;\times \;10^{7}s^{-1}$, are determined.

**Figure 4:**
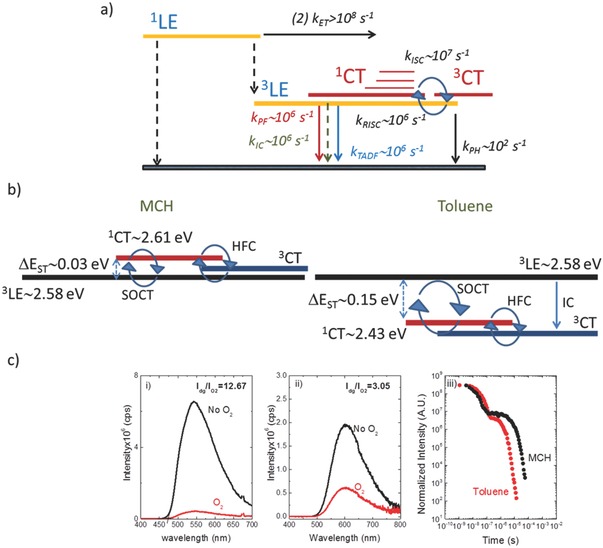
a) Energy diagram with a summary of the excited state dynamics of **DPTZ‐DBTO2** in MCH. b) Schematic representation of the electronic states involved in the RISC mechanism in **DPTZ‐DBTO2** in MCH and toluene; SOCT and HFC represent spin orbit coupling charge transfer and hyperfine coupling respectively. c) **DPTZ‐DBTO2** steady state emission in degassed and aerated conditions i) MCH, ii) toluene, iii) **DPTZ‐DBTO2** emission decays in MCH and toluene.

The reverse intersystem crossing rate from the ^3^LE to ^1^CT is determined from the TADF decay time, $\tau_{\mathrm{TADF}}=5.48\;\pm \;0.3\;\mu s$, and the amplitude ratio of the DF and PF decays obtained in Figure 2, $k_{\mathrm{RISC}}=\frac{I_{\mathrm{DF}}}{I_{\mathrm{PF}}}\;\frac{1}{\tau_{\mathrm{TADF}}}=(1.9\;\pm \;0.1)\;\times \;10^{6}s^{-1}$. Moreover, for small singlet–triplet Δ*E*_ST_ energy splitting, the two isoenergetic intersystem crossing rate constants, $k_{\mathrm{ISC}}^{S}$ and $k_{\mathrm{RISC}}^{0}$, Figure 1a, can be assumed to be very similar, and the energy barrier for the reverse intersystem crossing is then easily estimated from $\Delta E_{\mathrm{ST}}=-k_{B}T\ln\left(\frac{k_{\mathrm{RISC}}}{k_{\mathrm{RISC}}^{0}}\right)$ giving Δ*E*_ST_ = 0.08 ± 0.01 eV, in excellent agreement with the energy gap between the ^1^CT and ^3^LE states, ≈0.03 eV, estimated from the ^1^CT fluorescence and ^3^LE phosphorescence (Figure 2 and Figure 3). An energy diagram is shown in **Figure** **4**a, to summarize the excited state dynamics of **DPTZ‐DBTO2** in MCH.

In toluene solution, due to the higher polarity of this solvent, the ^1^CT shifts to lower energies (^1^CT_(toluene)_ ≈ 2.43 eV), Figure 4b. However, the ^3^LE energy is not significantly altered because this is a state with no charge transfer character, being a local excitonic triplet state. Therefore, in toluene the ^1^CT state lies below the local triplet, ^3^LE, and the energy gap between these two states increases to ≈0.15 eV. This simple change has significant implications in the RISC mechanism, and explains why the TADF contribution to the overall emission is so strongly affected by the polarity of the host. In MCH the TADF contributes 91% to the overall emission, while in toluene the TADF contribution is around 67%. A similar conclusion is drawn from the time resolved emission decays in both solvents, Figure 4c, where it is clear that the TADF contribution is larger in MCH than in toluene. Therefore, controlling the dielectric constant of the host matrix, as well as the energy ordering of its electronic states, is key for the TADF contribution in **DPTZ‐DBTO2**.

Basically, there are two mechanisms for interconversion between singlet and triplet states: spin orbit coupling (SOC) and hyperfine coupling (HFC). However, in systems where the D‐A electron coupling is weak, i.e., the CT character is strong, as it is in D‐A systems where the D‐A dihedral angle is close to 90°, as in **DPTZ‐DBTO2**, SOC is not operative between the ^1^CT and ^3^CT states, because the orbitals involved in both states are the same and thus the matrix element vanishes, ${〈}^{1}\mathrm{CT}|H_{\mathrm{SOC}}{|}^{3}\mathrm{CT}〉=0$. This process is thus formerly forbidden for the HOMO perpendicular to LUMO and no change in spatial orbital would occur upon interconverting between these two states, and so no orbital angular momentum change, as required for a spin flip, could occur.^14^ However, orthogonal D and A do allow efficient crossing between the ^1^CT state and an energetically close local triplet state, the ^3^LE state in this case, via a spin orbit charge transfer ISC process, (SOCT), as this spin flip transition couples to a change in orbital angular momentum (much like an *n‐*π* transition), concomitant with the measured high ISC rate.^15^ Therefore in **DPTZ‐DBTO2**, the ISC due to SOCT involves the ^1^CT and ^3^LE states, and thus achieving a very small energy gap between ^3^LE and ^1^CT is key for efficient thermally activated RISC. Consequently, for most well stabilized D‐A CT states, TADF is mediated between the ^1^CT and an energetically close local triplet state, ^3^LE.

Hyperfine coupling can, in principle, interconvert ^1^CT to ^3^CT and back,^16^ followed by internal conversion from ^3^CT to ^3^LE. However, ISC due to weak hyperfine interactions can only occur when the exchange energy, i.e., approximately the energy difference between ^1^CT and ^3^CT states, is smaller than the typical hyperfine energy of a large organic system, typically 1 to 20 μeV. For intramolecular charge transfer states of twisted conformation between the D and A chromophores, HFC between ^1^CT and ^3^CT might be operative, because the energy separation between ^1^CT and ^3^CT states of highly twisted D‐A molecules might be of this magnitude, but as can be seen from Figure 1b, the angle would need to be very close to 90°. Both HFC and SOCT have been proposed to be operative in TADF molecules showing enhanced device quantum efficiencies.[[qv: 16b]] However, this is still an open question and further investigation is needed to evaluate the role of HFC in the TADF mechanism. In the case of **DPTZ‐DBTO2**, the pronounced decrease in the TADF contribution in toluene when compared with MCH, indicates that the RISC mechanism is (at least) strongly dominated by SOCT between ^1^CT and ^3^LE.

Even assuming that HFC may be operative, and then in MCH, the underlying ^3^LE state is populated directly from ^1^CT due to SOCT, and perhaps by HFC from ^1^CT to ^3^CT followed by IC between ^3^CT and ^3^LE. RISC then occurs from ^3^LE to ^1^CT to originate TADF. In toluene, however, where the energy ordering is reversed, see Figure 4, several scenarios could be conceived, activated SOCT between ^1^CT to ^3^LE, followed by IC from ^3^LE to the ^3^CT, and HFC, occurring from ^1^CT to ^3^CT, populate the underlying ^3^CT state, instead of ^3^LE. TADF has then to be driven by RISC from ^3^CT and ^1^CT. So, in this case, if HFC were to give efficient spin interconversion within the charge transfer manifold of **DPTZ‐DBTO2**, no decrease in TADF contribution would be observed, since the entire triplet population arriving at ^3^CT would be converted back to ^1^CT. However, we observe a significant decrease in TADF contribution in toluene, which means that a far larger percentage of the triplet states are lost, and that can only be due to the smaller efficiency of the HFC mechanism between ^1^CT and ^3^CT. This result is easy to rationalize, given the fact that the HFC mechanism is efficient only for very small (<20 μeV) energy separations between ^1^CT and ^3^CT states. Therefore, we can assume that HFC is not operative at all, and then the drop in TADF contribution observed in toluene, is easily explained by the fact that the SOCT between ^1^CT and ^3^LE is an activated process in toluene, and since the energy gap between ^1^CT and ^3^LE is larger in toluene (Δ*E*_ST_ = 0.15 eV), compared with 0.03 eV in MCH, the TADF contribution will decrease, as the Boltzmann term is much smaller, but more initial prompt ^1^CT should be observed, as the longer PF decay time indicates in Figure 4c. Moreover, the ^3^LE has an additional fast decay channel open to it; as well as decaying directly to the ground state and ISC to feed ^1^CT, it may deactivate to the ^3^CT state by IC, which is not present in MCH. This process accelerates the decay of the ^3^LE state and so may explain a faster TADF decay in toluene as it is observed in Figure 4c. This beautifully explains the enhanced TADF observed at RT in MCH, and also why the TADF intensity decreases in more polar toluene, since with increasing polarity the energy gap between ^3^LE and ^1^CT increases.

This leads to the conclusion that the energy difference between the emissive ^1^CT state and the local triplet state, in this case ^3^LE, is key to achieving TADF with high efficiency. Therefore, the design of new TADF emitters should target minimizing the energy gap between the emissive ^1^CT state and any underlying local triplet states, instead of trying to minimize the gap between the ^1^CT and ^3^CT. However, the restriction imposed by the D–A molecular geometry in order to obtain efficient TADF emission, shows that even if ^1^CT and ^3^LE are almost degenerate RISC is only possible if the D–A relative orientation is allowed to rock about perfect orthogonality. We believe this rationale will open novel strategies to design TADF emitters. It should be noted that it is not really important whether the ^3^LE is above or below the ^1^CT state, providing there is a local triplet excited state in the proximity of the ^1^CT state, i.e., with an energy separation that is less than the width of the Boltzmann distribution, so thermally activated ISC between the two states can be efficient. However, the mechanism sustaining the intersystem crossing is important here. Since SOCT is not active between ^1^CT and ^3^CT states, the intersystem crossing between these two states can only occur by hyperfine coupling, and this mechanism is only active for very small energies (less than a few μeV). Therefore, it is vital that a local triplet state exists in the proximity of ^1^CT state so that intersystem crossing by SOCT can occur to support efficient TADF.

Direct CT absorption is also observed in solution, at 10^−2^ m concentration, as a small band on the foot of the donor ππ* absorption edge, having an extinction coefficient, *ε* ≅ 10^2^ m ^−1^ cm^−1^, concomitant with a very low transition dipole moment. Measurements of phenothiazine at the same concentrations reveal no such band and so it arises as a direct result of the coupling with the acceptor unit in **DPTZ‐DBTO2**, (Figure S14, Supporting Information). Aggregation effects were shown not to be the cause of this new band.

**Figure 5:**
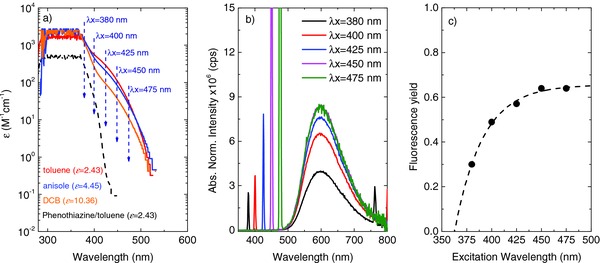
a) Molar extinction coefficients of **DPTZ‐DBTO2** obtained in solvents with increasing dielectric constant, showing a blueshift indicative of an nπ* transition, in panel (5a) the spectra are represented in a log scale to highlight the differences to the phenothiazine donor absorption, and the wavelengths used to selectively excite the new absorption band are indicated. b) Fluorescence emission spectra of **DPTZ‐DBTO2** in toluene solution obtained in front‐face geometry, with increasing excitation wavelength and normalized to the light absorption at the excitation wavelength. c) For direct excitation of the nπ* into the CT state, the fluorescence yield in MCH is as high as 0.6.

The new band was found to show a blueshift with increasing solvent polarity, indicative of the transition having nπ* character. This is shown in **Figure** **5**a. Moreover, selective excitation of this new band leads to emission from the ^1^CT state, with identical spectra to those observed when directly exciting the donor of **DPTZ‐DBTO2**. However, as the excitation wavelength was increased to excite more preferentially this new transition, the emission intensity also increased, Figure 5b. In this case the PLQY in MCH solution was estimated to increase to ≈0.6: details are given in Figure S15 (Supporting Information). Because this new transition is present only in **DPTZ‐DBTO2**, and it is very weak we ascribe it to a direct CT absorption band, which avoids the population of ^1^LE excitonic states. Importantly, this indicates that nonradiative decay from ^1^LE presents a significant loss mechanism when the donor ^1^LE state is optically excited, and that by avoiding this far higher PLQY can be achieved, see Figure 5c.

### 2.3 Photophysics in Solid Hosts

When dispersed in a solid zeonex matrix the **DPTZ‐DBTO2** emission retains the same CT character observed in MCH and toluene solutions. The luminescence spectrum appears broad and devoid of any structure, peaking at 560 nm. Again the ^1^CT fluorescence is observed to be strongly oxygen dependent even in solid film. The triplet yield is determined from these measurements in good agreement with the previous determinations in MCH as 81 ± 5%.

**Figure 6:**
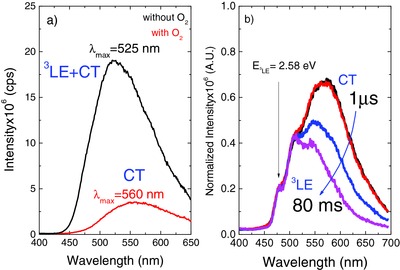
a) Steady‐state emission spectra of **DPTZ‐DBTO2** in zeonex solid thin film, at room temperature. b) Time resolved emission spectra of **DPTZ‐DBTO2** in zeonex at 20 K, normalized on the ^3^LE phosphorescence at 475 nm.

Noticeably, in zeonex the room temperature oxygen‐free spectrum of **DPTZ‐DBTO2** shows a striking contribution from ^3^LE phosphorescence, causing the peak and onset of the emission to effectively blueshift from 560 to 525 nm, even at RT, see **Figure** **6**a. In aerated conditions the prompt fluorescence from the ^1^CT state peaks at 560 nm. When degassed the emission peak blueshifts to 525 nm and the intensity significantly increases due to the contribution of both TADF and ^3^LE‐phosphorescence. RT‐phosphorescence has been previously observed in our other D–A–D materials.^[2b,8]^ Phosphorescence from ^3^LE is even more clearly observed at low temperature, Figure 6b. The TADF appearing from the ^1^CT state rapidly decays in the μs time range, leaving behind the long‐lived and blueshifted ^3^LE phosphorescence, showing a longer lifetime (80 ms) than the ^1^CT emission, which again is in excellent agreement with that of phosphorescence from *N*‐substituted phenothiazines,^6^ and indicates that the ^3^LE state has predominantly ^3^(nπ*) character^12^ commensurate with a strongly phosphorescent state.

Fluorescence emission from the ^1^LE state in **DPTZ‐DBTO2** is also observed at early times following excitation in solid matrix, decaying in 6 ns, and matching the phenothiazine fluorescence (^1^D), (Figure S16, Supporting Information). These observations are of crucial importance, because they fundamentally show that the ^1^LE decay to the ground state and ISC to the ^3^LE states is able to compete with the population of the ^1^CT state, which can be attributed to the near perpendicular D‐A structure of the **DPTZ‐DBTO2** molecule and the rigid environment.

The intramolecular TADF origin of the delayed fluorescence observed in **DPTZ‐DBTO2** films in zeonex is again confirmed by the strictly linear dependence of the delayed fluorescence integral with excitation intensity (Figure S17, Supporting Information). Moreover, the temperature dependence of the **DPTZ‐DBTO2** delayed fluorescence, (Figure S18, Supporting Information), shows a pronounced increase of the emission intensity with temperature from 20 to 250 K, but remains practically constant above 250 K, due to the very small ^1^CT–^3^LE energy barrier, determined as 0.02 eV in excellent agreement with singlet–triplet energy gap.

**Figure 7:**
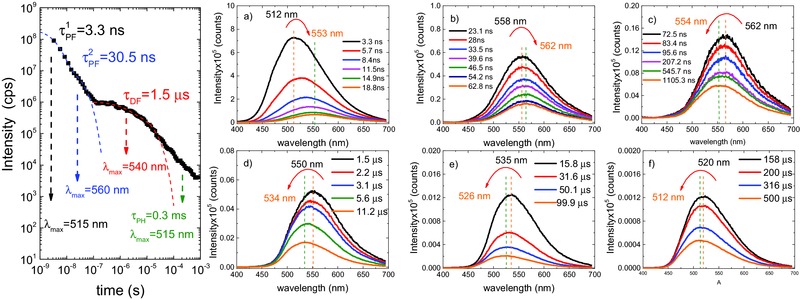
Emission decay and time resolved spectra obtained at different delay times for **DPTZ‐DBTO2** dispersed in zeonex at RT. a) ^1^CT, prompt fluorescence emission spectra showing a contribution from ^1^LE fluorescence at very early times (less than 3 ns). b) ^1^CT fluorescence, possibly also containing a DF contribution, emitting around 560 nm between 23 and 63 ns. c) From 73 ns to 1.1 μs, the ^1^CT emission is dominated by delayed fluorescence, and at later times the contribution of the far more slowly decaying ^3^LE phosphorescence emerges, causing a blueshift in the emission peak. d) ^1^CT delayed fluorescence, decaying from 1.5 to 11.2 μs. The relatively stronger contribution of the underlying ^3^LE phosphorescence causes the emission maxima to effectively shift to shorter wavelengths. e,f) ^1^CT delayed fluorescence, decaying from 15.8 to 500 μs, showing a continuous blueshift in the maxima toward 505 nm where the ^3^LE phosphorescence peaks.

Despite the small energy barrier for triplet harvesting that is observed for **DPTZ‐DBTO2** dispersed in zeonex, the emission decay is more complex than that observed in solution due to the presence of short‐lived ^1^LE fluorescence and long‐lived ^3^LE phosphorescence that overlap with the ^1^CT emission, see **Figure** **7**.

In a zeonex matrix the prompt emission is described by two decay regimes, a fast component of around 3.3 ns, corresponding to a blueshifted emission peaking at ≈512 nm (2.42 eV), which we assign to the fluorescence emission of the ^1^CT state strongly mixed with ^1^LE fluorescence. This emission progressively shifts to longer wavelengths, from 512 to 560 nm (2.21 eV), due to the growing contribution of the ^1^CT emission, decaying with a 30 ns time constant. The delayed fluorescence, also from the ^1^CT state, decays with a time constant of 1.5 μs, the emission peak being observed at 560 nm up to a delay time of ≈400 ns. It then progressively shifts back to shorter wavelengths, due to the growing contribution of the underlying ^3^LE phosphorescence. For delay times in the μs time range, the delayed emission maximum is at 540 nm (2.29 eV), and at late times, into the ms time range, the emission peaks at 512 nm, closely matching the peak of the ^3^LE phosphorescence.

This behavior of **DPTZ‐DBTO2** in solvent‐free rigid matrix can be understood from the basic equation governing electron transfer, *K*_ET_ = 4π^2^/*hV*_DA_^2^ [FCWD],[[qv: 13a]] where *V*_DA_ is the electronic D–A coupling, which has been minimized in **DPTZ‐DBTO2** by the near‐orthogonality of the D and A fragments, and the FCWD is the Frank–Condon weighted density of states, the measure of the total spatial overlap of all D and A vibrational modes. In the case of **DPTZ‐DBTO2** where D and A are nearly perpendicular, only out‐of‐plane modes can thus couple to enable state crossing, also causing the FCWD to be rather small. Thus, in rigid matrix one can see that the initial intramolecular electron transfer will be extremely slow. This is why at very early times ^1^D emission from the donor phenothiazine fragment is observed in rigid medium (Figure S16, Supporting Information).

The excited state dynamics of **DPTZ‐DBTO2** excited at 355 nm when dispersed in a 4,4‐*bis*(9‐carbazolyl)‐1,1‐biphenyl (CBP) matrix follow a similar trend to that seen for **DPTZ‐DBTO2** dispersed in zeonex films, (Figure S19, Supporting Information). From these measurements we are able to fully describe the **DPTZ‐DBTO2** excited state dynamics: Upon optical excitation of the phenothiazine donor (^1^LE), and depending on the environment, the ^1^CT state is formed by relatively fast (in solution), or slower (in solid films), electron transfer. In the solid state, initial fluorescence from the ^1^LE state is clearly observed. Intersystem crossing from ^1^LE to ^3^LE occurs in competition with the population of the ^1^CT state, which emits at 560 nm. Equilibrium between ^1^CT and ^3^LE states is achieved through the recycling of CT singlet and triplet states, via intersystem crossing and thermally assisted reverse intersystem crossing, yielding the long‐lived thermally assisted delayed fluorescence from ^1^CT. At late times phosphorescence from ^3^LE is observed.

### 2.4 Device Physics

**Figure 8:**
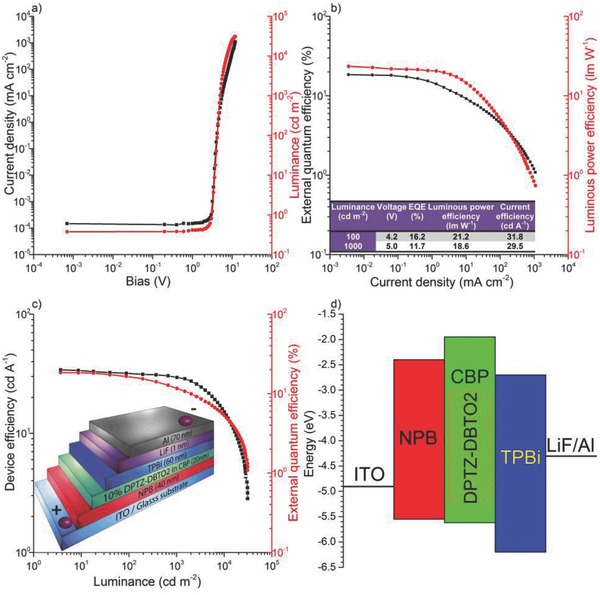
**DPTZ‐DBTO2**:CBP OLED characteristics. a) Current density and luminance versus bias. b) EQE and luminous power efficiency versus current density, inset, characteristics of devices at 100 and 1000 cd m^−2^ brightness. c) Device efficiency and EQE versus luminance, inset, device structure showing layer thicknesses. d) Device structure in energetic domain as a representation of HOMO–LUMO levels of the respective layers.

**Figure** **8** shows that very simple unoptimized devices containing **DPTZ‐DBTO2** as the emitter in CBP host (architecture: ITO/NPB (40 nm)/10% DPTZ‐DBTO2:CBP (20 nm)/TPBi (60 nm)/LiF/Al), where NPB is N,N′‐Di(1‐naphthyl)‐N,N′‐diphenyl‐(1,1′‐biphenyl)‐4,4′‐diamine, give excellent performance with EQE of 18.8% (at 10 cd m^−2^ luminance with no external outcoupling enhancement). Roll‐off is seen at high brightness, consistent with many other TADF devices utilizing simple emitter layer architectures,[[qv: 1a]][[qv: 1b]] decreasing the EQE value to 16.2% at 100 cd m^−2^ and 11.7% at 1000 cd m^−2^.

In devices, whereas the hole injection onto the HOMO of the donor is energetically favorable, the phenothiazine LUMO level is far too shallow, <−1.9 eV (from our measurements) compared to that of the dibenzothiophene‐*S,S*‐dioxide acceptor, −3.0 eV (see electrochemical data, Figure S6, Supporting Information), so electron injection will occur onto the decoupled A unit not the phenothiazine. Therefore, charges will be injected directly into the donor fragment HOMO and acceptor fragment LUMO of **DPTZ‐DBTO2**. Thus excited state formation from charge recombination directly generates the CT charge transfer states, not the unrelaxed local singlet and triplet states. This is well documented in previous work on interfacial exciplex devices^17^ and also gives rise to the very low turn on voltages observed here and in other TADF devices.^18^ This then avoids losses associated with the excitonic states localized on the D and A fragments, contributing to a much higher effective PLQY, as confirmed by direct absorption to the ^1^CT state. We also note that the **DPTZ‐DBTO2** emitter is also within a rigid CBP matrix which may also help to give a high PLQY. All ^3^CT states formed on charge recombination will rapidly internally convert to a near isoenergetic ^3^LE state; probably this is why the ^3^CT state can never be resolved spectroscopically, however, these triplet states are then recycled by RISC to the ^1^CT state and emit TADF.

## 3 Conclusions

This investigation has focused on a TADF emitter with D–A–D subunits with near orthogonal orientation to study the interplay between the excited states localized on the D and A subunits and the CT excited states in the TADF mechanism. We have established that SOCT between ^1^CT and the underlying local triplet state, ^3^LE, is the dominant ISC mechanism in **DPTZ‐DBTO2**. It is crucial to design the correct CT geometry into the ground state of the emitter, thereby guaranteeing that CT formation always occurs even in a nonpolar rigid matrix or device host, and that an underlying local triplet excited state, nearly degenerate with the emissive ^1^CT state, exists to facilitate TADF, and that this energy gap is the true barrier to RISC.

Weak, direct absorption to the ^1^CT state is clearly observed in **DPTZ‐DBTO2**, showing that initial photoexcitation to the donor singlet state introduces a major nonradiative loss channel. However, in OLEDs, charge recombination into the HOMO of the donor and LUMO of the acceptor that directly forms CT states on the emitter, avoids this loss associated with the local excitonic states, thereby giving rise to higher device efficiencies, along with the rigidity of the matrix which will also minimize nonradiative decay.

Other TADF emitters with near perpendicular D‐A structure have been reported recently with slightly different molecular structures.^19^ However, in most of these molecules lower device efficiencies have been obtained, when compared with the EQEs that are achieved with **DPTZ‐DBTO2** devices. Despite the fact that some of these emitters may share common photophysics with **DPTZ‐DBTO2**, reasons for low device EQEs can be numerous, including material purity, device fabrication, ordering of energy levels, etc., and it is in general difficult to establish definite reasons to explain the observation of lower EQE in devices fabricated with other, even if similar, molecular structures. However, from this work it is clear that the precise ordering and energy splitting between ^1^CT and ^3^LE states in **DPTZ‐DBTO2** and the D–A molecular geometry are critical in order to avoid fully rigid and orthogonal geometry between D–A moieties.

## 4 Experimental Section

Solution measurements used concentrations in the 10^−5^–10^−2^ m range, and samples were degassed using 5 freeze/thaw cycles. **DPTZ‐DBTO2**/zeonex films were prepared by spin coating with **DPTZ‐DBTO2**:zeonex ratio of (1:20 w/w). **DPTZ‐DBTO2**/CBP films were prepared by coevaporation. Absorption and emission spectra were collected using a UV‐3600 double beam spectrophotometer (Shimadzu), and a Fluorolog fluorescence spectrometer (Jobin Yvon). Phosphorescence, prompt fluorescence, and delayed emission (DF) spectra and decays were recorded using nanosecond gated luminescence and lifetime measurements (from 400 ps to 1 s) using either a high energy pulsed Nd:YAG laser emitting at 355 nm (EKSPLA) or a N_2_ laser emitting at 337 nm. Emission was focused onto a spectrograph and detected on a sensitive gated iCCD camera (Stanford Computer Optics) having subnanosecond resolution. PF/DF time resolved measurements were performed by exponentially increasing gate and delay times; details can be found elsewhere.^20^

OLED devices were fabricated using precleaned indium‐tin‐oxide (ITO) coated glass substrates purchased from Ossila with a sheet resistance of 20 Ω cm^−2^ and ITO thickness of 100 nm. The OLED devices had a pixel size of 2 × 1.5 mm. The small molecule and cathode layers were thermally evaporated using the Kurt J. Lesker Spectros II deposition apparatus at 10^−6^ mbar. All organic materials and aluminum were deposited at a rate of 1 Å s^−1^, the **DPTZ‐DBTO2**:CBP layer was deposited by coevaporation: 0.2 Å s^−1^ for **DPTZ‐DBTO2** and 2 Å s^−1^ for CBP; the LiF layer was deposited at 0.1 Å s^−1^.

## Supporting information

As a service to our authors and readers, this journal provides supporting information supplied by the authors. Such materials are peer reviewed and may be re‐organized for online delivery, but are not copy‐edited or typeset. Technical support issues arising from supporting information (other than missing files) should be addressed to the authors.

Supplementary
