# Supplementary material for: The Role of Local Triplet Excited States and D‐A Relative Orientation in Thermally Activated Delayed Fluorescence: Photophysics and Devices
Source: Adv Sci (Weinh). 2016 Jul 18;3(12):1600080. doi: 10.1002/advs.201600080 (PMC5157178; doi:10.1002/advs.201600080)
Supplement: Supplementary file 1 — Supplementary [file ADVS-3-0-s001.pdf]

Copyright WILEY-VCH Verlag GmbH & Co. KGaA, 69469 Weinheim, Germany, 2016.

ADVANCED SCIENCE
Open Access

Supporting Information

for *Adv. Sci.*, DOI: 10.1002/advs.201600080

The Role of Local Triplet Excited States and D-A Relative Orientation in Thermally Activated Delayed Fluorescence: Photophysics and Devices

Fernando B. Dias, Jose Santos, David R. Graves, Przemyslaw Data, Roberto S. Nobuyasu, Mark A. Fox, Andrei S. Batsanov, Tiago Palmeira, Mário N. Berberan-Santos, Martin R. Bryce, and Andrew P. Monkman*

Copyright WILEY-VCH Verlag GmbH & Co. KGaA, 69469 Weinheim, Germany, 2013.

Supporting Information

The Role of Local Triplet Excited States and D-A Relative Orientation in Thermally Activated Delayed Fluorescence: Photophysics and Devices

Fernando B. Dias[1], Jose Santos[1,4], David Graves[1], Przemyslaw Data[1,5], Roberto S. Nobuyasu[1], Mark A. Fox[2], Andrei S. Batsanov[2], Tiago Palmeira[3], Mário N. Berberan-Santos[3], Martin R. Bryce[2], Andrew P. Monkman[1]*

**e-mail corresponding author: f.m.b.dias@durham.ac.uk*

SI 1. Computations (Geometry optimization, HOMO, LUMO and excited state, singlet and triplet, energies, simulated absorption spectra).

All geometry optimizations of **DPTZ-DBTO2**, phenothiazine and dibenzothiophene-*S*,*S*-dioxide were carried out at B3LYP[S1]/6-31G(d) [S2] using the GAUSSIAN09[S3] package and confirmed as true minima by frequency calculations. Electronic structure (molecular orbital) data were obtained at the hybrid-DFT B3LYP/6-31G(d) with molecular orbitals plotted using GABEDIT[S4] and orbital contributions determined with GAUSSSUM.[S5] TD-DFT computations on the S_0 optimized geometries at B3LYP/6-31G(d) were used to determine the nature and energies of the singlet and triplet excited states.

Three minima (*A*, *B* and *C*) of **DPTZ-DBTO2** were obtained with relative energies of only 0.6 kcal mol^{-1} between them. Geometry *A* of C_{2v} symmetry is the same conformer as the X-ray structure, while *B* also of C_{2v} symmetry has both phenothiazinyl groups folded in opposite directions to those in *A*. Geometry *C* has two non-equivalent phenothiazinyl groups thus is of C_s symmetry. The energy barrier to planarity in unsubstituted phenothiazine is only 1.7 kcal

---

[S1] a) Becke, A.D., Density-functional thermochemistry. 3. The role of exact exchange. J. Chem. Phys. **98**, 5648–5652 (1993); b) Lee, C., Yang, W., Parr, R. G., Development of the Colle-Salvetti correlation energy formula into a functional of the electron-density. Phys. Rev. B **37**, 785–789 (1988).

[S2] a) Petersson, G. A., Al-Laham, M. A., A complete basis set model chemistry .2. Open-shell systems and the total energies of the 1st-row atoms. J. Chem. Phys. **94**, 6081–6090 (1991); b) Petersson, G. A., Bennett, A., Tensfeldt, T. G., Al-Laham, M. A., Shirley, W. A., Mantzaris, J., A complete basis set model chemistry .1. Open-shell systems and the total energies of the 1st-row atoms and hydrides of the 1st row elements. J. Chem. Phys. **89**, 2193–2218 (1988).

[S3] GAUSSIAN09, Revision A.02, M. J. Frisch et al, *Gaussian, Inc.*, Wallingford CT, 2009.

[S4] Allouche, A. R., Gabedit-A Graphical User Interface for Computational Chemistry Softwares. J. Comput. Chem. **32**, 174–182 (2011).

[S 5] O'Boyle, N. M., Tenderholt, A. L., Langner, K. M., cclib: A library for package-independent computational chemistry algorithms. J. Comput. Chem. **29**, 839–845 (2008).

mol^{-1} (0.07 eV) so all conformers *A*-*C* of **DPTZ-DBTO2** would be expected to be present in solutions.

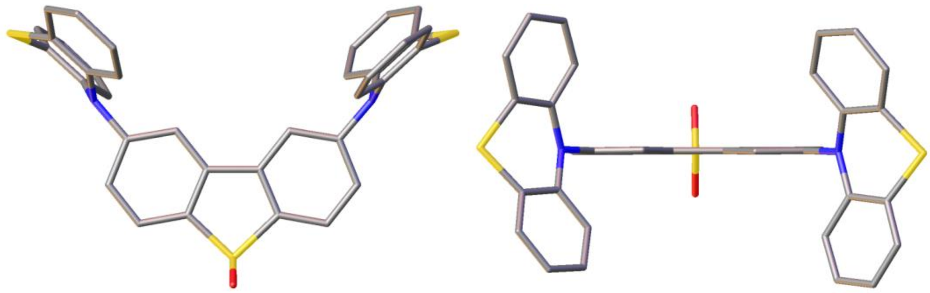

Figure S1-Views of geometry *A* for **DPTZ-DBTO2** showing nearly perpendicular orientations between donor and acceptor units.

All three conformers, *A*-*C*, of **DPTZ-DBTO2** give similar computed energies of excited states and molecular orbitals. The only notable difference between the three is that the degenerate HOMO and HOMO+1 differ in geometry *C* due to non-equivalent phenothiazinyl groups.

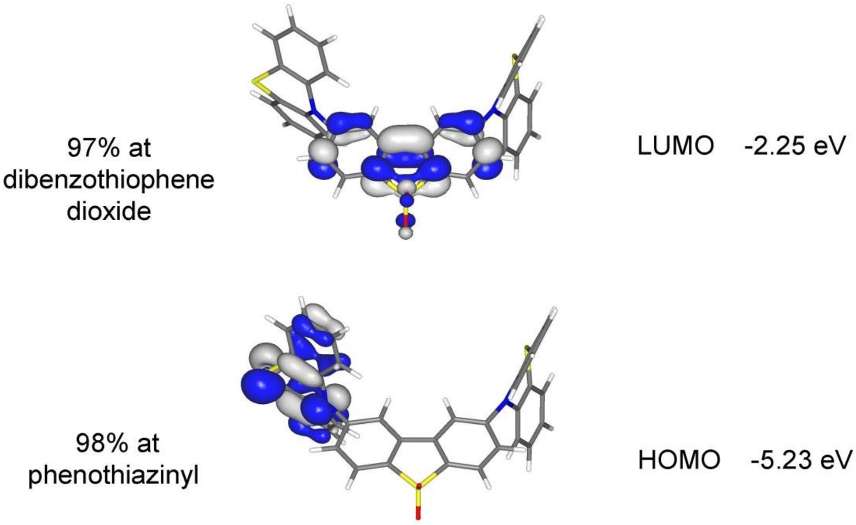

Figure S2-Frontier orbitals and relative orbital contributions (%) of geometry *C* for **DPTZ-DBTO2**.

For comparison with **DPTZ-DBTO2**, the frontier orbitals of phenothiazine and dibenzothiophene-*S*,*S*-dioxide were also examined. The LUMO energy in dibenzothiophene-*S*,*S*-dioxide is -1.81 eV whereas the HOMO energy in phenothiazine is -5.00 eV. The HOMO and LUMO in **DPTZ-DBTO2** are thus essentially localized in the donor and acceptor units respectively. The energy difference of 0.44 eV in the LUMO energy between **DPTZ-DBTO2** and dibenzothiophene-*S*,*S*-dioxide is almost double the difference of 0.23 eV in the HOMO energy between **DPTZ-DBTO2** and phenothiazine. This is expected as the acceptor unit has two ‘substitutions’ whereas the donor unit has one.

TD-DFT computations on the ground state (S_0) minima *A*-*C* of **DPTZ-DBTO2** show that both singlet (S_1, 2.46 eV) and triplet (T_1, 2.45 eV) states are very close in energy. The transitions (HOMO $\rightarrow$ LUMO) in $S_0 \rightarrow S_1$ and $S_0 \rightarrow T_1$ are both charge transfer.

Simulated spectra for dibenzothiophene-*S*,*S*-dioxide, phenothiazine and DPTZ-DBTO2. Figure 1 shows that the simulated absorption spectrum of **DPTZ-DBTO2** may be viewed as the sum of the simulated spectra of dibenzothiophene-*S*,*S*-dioxide and phenothiazine. Figure 2 shows the lowest bands predicted in the 325 to 550 nm region. All bands above 380 nm have no oscillator strengths (f = 0.0000).

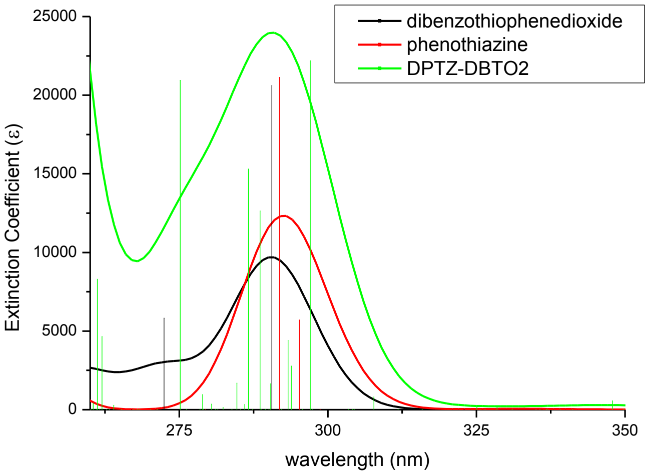

Figure S3. Simulated absorption spectra of dibenzothiophenedioxide, phenothiazine and **DPTZ-DBTO2**. The vertical lines are from singlet state data from TD-DFT computations and the bands are simulated using a half-height width of 0.12 eV. The extinction coefficients are calculated from the oscillator strengths by 240000.

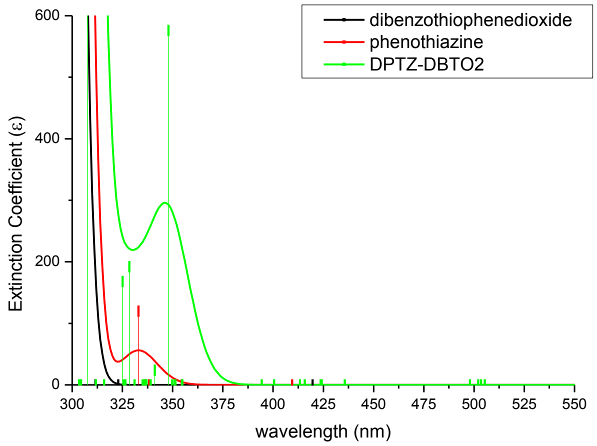

Figure S4. Simulated absorption spectra of dibenzothiophenedioxide, phenothiazine and **DPTZ-DBTO2** in the 300 to 550 region. The points marked along the wavelength axis are predicted values with zero oscillator strengths.

Effect of C-C-N-C torsion angles on simulated absorption spectra of DPTZ-DBTO2.

Figure S3 shows the increase in oscillator strengths of the charge transfer bands as orientations deviate from the near orthogonal and most stable minimum of **DPTZ-DBTO2**. The geometries with fixed 70º and 90º C-C-N-C torsion angles are higher than the most stable minimum by 0.018 eV and 0.007 eV, respectively. It should be pointed out here that the 70º and 90º geometries are similar as the corresponding optimized C-C-N-C angles are at 89.8º and 76.6º, respectively, due to the fold present in each phenothiazinyl group. The oscillator strengths are still small in these less orthogonal geometries with a maximum of only f = 0.0022 for the lowest singlet charge transfer (^{1}CT) arising from the 70º geometry.

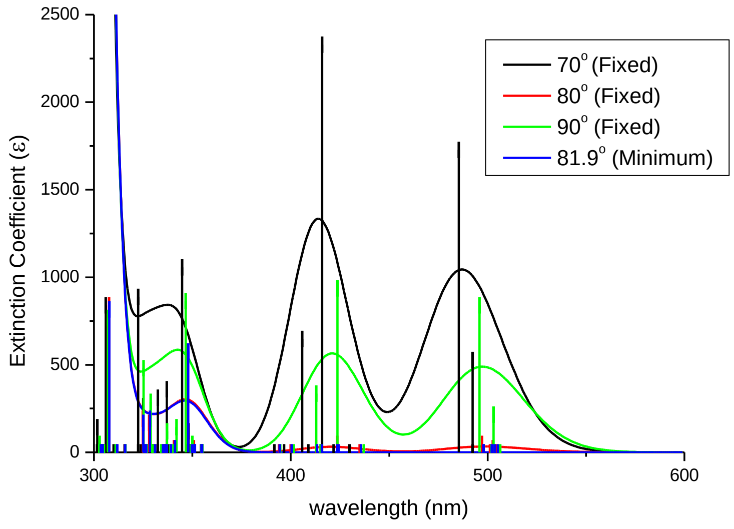

Figure S5. Effect of the phenothiazinyl group orientations on the simulated spectra from optimized geometries of **DPTZ-DBTO2** with fixed C-N-C-C torsion angles.

SI 2. Electrochemical Studies (HOMO, LUMO energies).

Cyclic voltammetry measurements were conducted in 1.0 mM concentrations of all molecules. Electrochemical studies were carried out in 0.1 M solutions of Bu_4NBF_4, 99% (Sigma Aldrich) in dichloromethane (DCM) solvent, CHROMASOLV®, 99.9% (Sigma Aldrich) at room temperature. The electrochemical cell comprised of 1 mm diameter disk platinum as a working electrode, an Ag/AgCl electrode as a reference electrode and a platinum coil as an auxiliary electrode.

Cyclic voltammetry measurements were conducted at room temperature at a potential rate of 50 mV/s and were calibrated against a ferrocene/ferrocenium redox couple. Compounds were characterized by CV to determine the ionization potential (IP) and the electron affinity (EA) values, which were obtained from the potentials of the onset of the redox peaks, provided that these potentials are expressed on the absolute potential scale, *i.e.* with respect to the vacuum level. The absolute potential of FcH/FcH^+ in nonaqueous electrolytes is 5.1 V.[S6] This leads to the following equations:

$$IP(eV) = |e|(E_{ox(onset)} + 5.1) \quad (1)$$

$$EA(eV) = -|e|(E_{red(onset)} + 5.1) \quad (2)$$

The HOMO-LUMO levels were determined electrochemically, from the CV data analysis described above, taking into consideration that EA and IP directly correspond to LUMO and HOMO energies respectively.[S7, S8]

The first *p*-doping redox couple of **DPTZ-DBTO2** as seen on the figure has slightly higher oxidation potential (0.23 V) than the redox couple of pure phenothiazine.

The first redox process of *n*-doping of **DPTZ-DBTO2** is localised over the dibenzothiophene-*S*,*S*-dioxide core. This redox couple occurs at a higher negative potential than for pure dibenzothiophene-*S*,*S*-dioxide by 0.18 V. Clearly the two phenothiazine units have a small influence on the electronic structure of the acceptor core and lower the EA (LUMO) energy value.

[S6] Cardona, W., Li, Kaifer, A. E., Stockdale, D. Bazan, G. C., Electrochemical Considerations for Determining Absolute Frontier Orbital Energy Levels of Conjugated Polymers for Solar Cell Applications, Adv. Mater. **23**, 2367-2371 (2011).

[S7] Data, P., Pander, P., Lapkowski, M., Swist, A., Soloducho, J., Reghu, R. R., Grazulevicius, J. V., Unusual properties of electropolymerized 2,7- and 3,6- carbazole derivatives. Electrochim. Acta **128**, 430-438 (2014).

[S8] J.-L. Bredas, Mind the gap!, Mater. Horiz. **1**, 17-19 (2014).

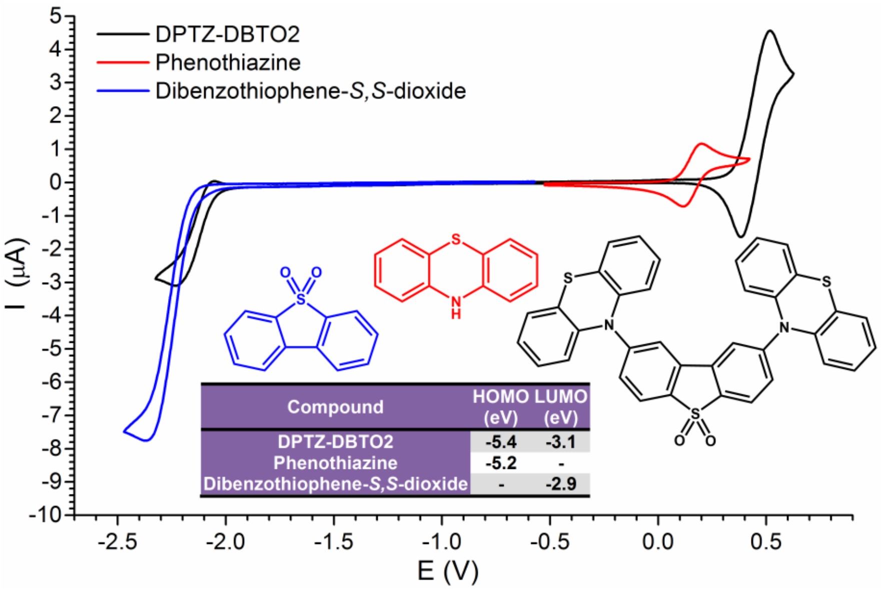

Figure S6- Cyclic voltammetry measurements and HOMO LUMO energies of **DPTZ-DBTO2**, Phenothiazine, and Dibenzothiophene-S,S-dioxide.

Whereas the phenothiazine donor is readily oxidizable, to our knowledge its reduction potential has not been reported in the literature; and we also were not able to observe a reduction wave in our electrochemical measurements, which places an upper boundary of -1.9 eV on the LUMO level. Further, from the measured HOMO level of phenothiazine and **DPTZ-DBTO2**, -5.4 eV, adding the energy of the ^{1}LE HOMO-LUMO transition, 3.45 eV, also gives an estimate of the phenothiazine LUMO level of -1.95 eV. Taking our experimental upper bound as a more conservative value for the LUMO, we also measure the LUMO of the *S,S*-dioxide acceptor unit at -2.9 eV, thus at least 1.05 eV below that of the donor. The computed LUMO energies for phenothiazine and the *S,S*-dioxide acceptor molecules are -0.02 and -1.81 eV, respectively. These DFT LUMO energies are generally overestimated as reported elsewhere,[S9] compared to experimental values, but the large energy difference of 1.8 eV between the two LUMO energies is in accord with CV measurements.
Given a typical ‘exciton binding’ energy of 0.5 eV then it is still not conceivable that the phenothiazine will be reduced in preference to the dibenzothiophene-*S,S*-dioxide unit. Further, the acceptor LUMO matches very well to the injection level of LiF and so very efficient injection of electrons from LiF into the acceptor fragment will result, leading to a very low turn on voltage as we observe in our devices.
A review of the literature reveals that the phenothiazinium cation can be reduced at very low pH levels (pH <2) where strong protonation of the molecular anion can stabilize the molecule to reduction. Clearly, our experiments are never in such strong acid environments in solution or within a device, thus we are sure that given the ease with which the dibenzothiophene-*S,S*-dioxide can be reduced, it will always accept the injected electron. For further information see ref S10.[S10]

[S9] Kabanda, M. M., Murulana, L. C., Ebenso, E. E., Int. J. Electrochem. Sci., **7**, 7179 – 7205 (2012).
[S10] Wardman, P., Reduction Potentials of one-electron couples involving free radicals in aqueous solution’ J. Phys. Chem. Ref. Data, **18**, 1637-1753 (1989).

This electrochemical data strongly supports the thesis that electrons are injected into the decoupled acceptor of **DPTZ-DBTO2** whereas holes are injected into the donor fragment and subsequent recombination within the emission layer directly creates CT states, not local excitonic states on the donor or acceptor fragment as a precursor to CT formation.

SI 3. Effect of oxygen on the DPTZ-DBTO2 steady state fluorescence

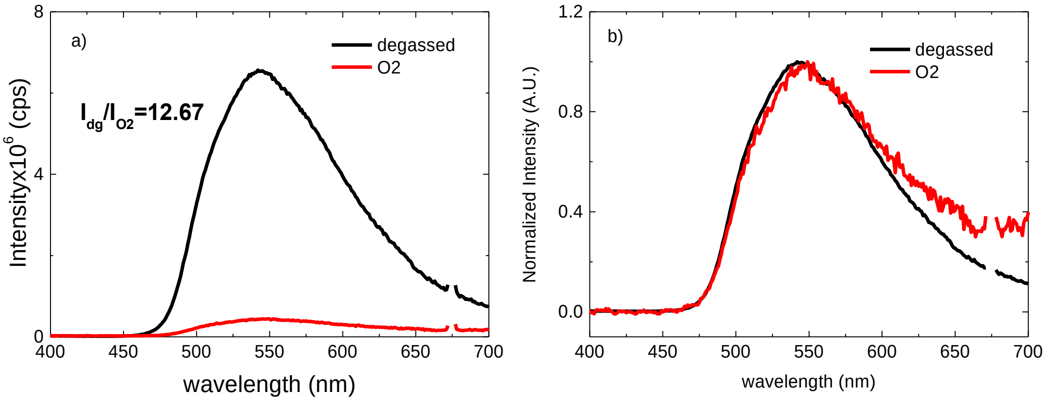

$$\frac{I_{SS}^{dg}}{I_{SS}^{O2}} - 1 = \frac{I_{DF}}{I_{PF}} = 11.7$$

Figure S7- a) A comparison is shown between aerated (O2, red) and degassed (black), steady-state fluorescence spectra of **DPTZ-DBTO2** in MCH solution at RT. Upon degassing the solution, the fluorescence integral increases by a factor of ca. 13. b) The emissions obtained in aerated and degassed solutions very closely match each other.

SI 4. Steady-state and delayed fluorescence spectra.

The delayed fluorescence (DF) from **DPTZ-DBTO2** in MCH solution, obtained after a time delay of 1 µs, and using an integration time of 100 µs, is compared with the steady-state emission. There is an excellent agreement between both spectra. Clearly, DF and steady-state fluorescence appear from the same excited state.

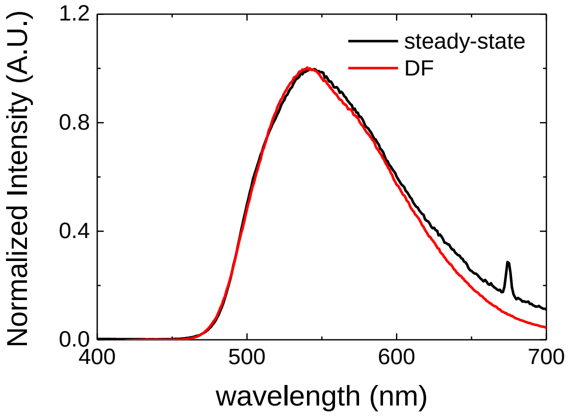

Figure S8-Steady-state fluorescence and integrated delayed fluorescence in MCH at RT.

SI 5. Intensity dependence of the DPTZ-DBTO2 delayed fluorescence in MCH solution. The intensity dependence of the **DPTZ-DBTO2** delayed fluorescence spectrum and emission integral are shown. The fit in b) is obtained with a strictly linear equation (with gradient 1), showing a correlation coefficient of 0.999. Spectra were acquired with 1μs delay time and integrated for 200 μs.

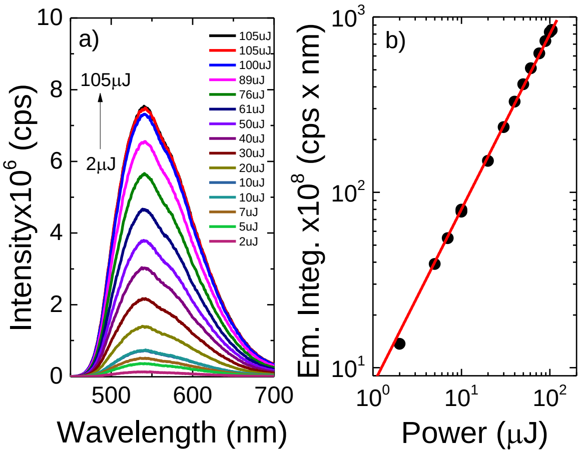

Figure S9- Intensity dependence of the DPTZ-DBTO2 delayed fluorescence in MCH solution.

SI 6. Bathochromic shift is observed in the fluorescence spectrum of DPTZ-DBTO2. Figure a) shows the absorption of **DPTZ-DBTO2** collected in solvents of increasing dielectric constant. No solvatochromic shift is observed in the main ππ* absorption bands of **DPTZ-DBTO2**. Contrary to the absorption, the emission spectrum of **DPTZ-DBTO2** is strongly affected by the solvent polarity. In non-polar MCH the emission peaks at 540 nm, with a Stokes shift value around 1.38 eV (190 nm). With increasing solvent polarity the emission further red shifts and its intensity decreases. Figure b) shows the **DPTZ-DBTO2** emission spectra in methylcyclohexane (MCH), toluene and chlorobenzene (PhCl), normalized for the absorption at 337 nm. In ethanol, the emission is completely quenched.

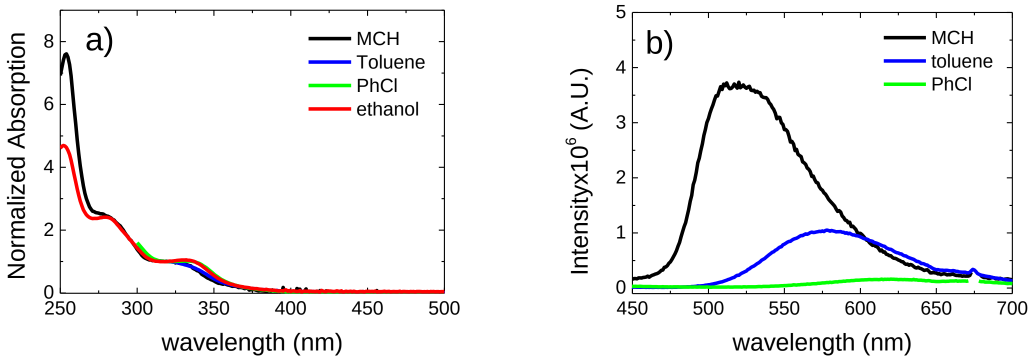

Figure S10-Bathochromic shift observed from methylcyclohexane to toluene in the fluorescence spectra of **DPTZ-DBTO2**.

SI 7. Emission decay in toluene at RT.

The figure shows plots of the **DPTZ-DBTO2** emission intensity decay over a time interval spanning 8 decades, obtained at RT in toluene. Prompt and delayed components are clearly separated in time, showing exponential decays. In toluene the best fit of the prompt fluorescence is obtained only with a sum of two exponential functions, with 21 ns and 52 ns lifetimes. This longer component may already include contribution from triplet states. The long component now decays with a 1.03 μs time constant.

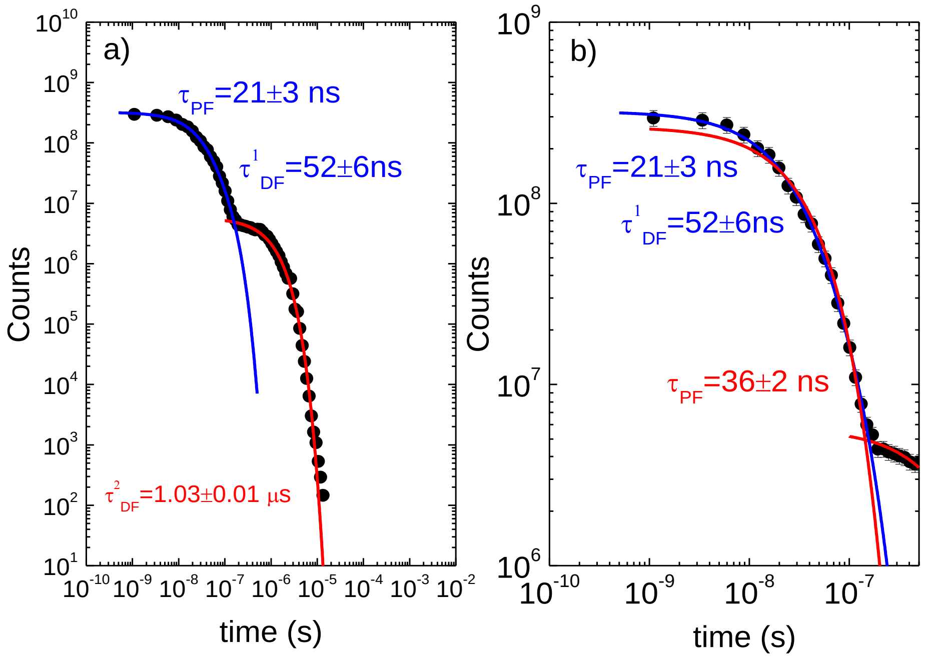

Figure S11-Emission decay in toluene at RT.

SI 8. Direct ground state absorption to the CT manifold in DPTZ-DBTO2.

In all measurements of the absorption of **DPTZ-DBTO2** under standard conditions we observed no trace of ‘direct’ ground state absorption to the CT manifold, such as seen in less orthogonal ICT molecules.[S11] However, in extremely high concentration solutions a new absorption band was observed at the foot of the donor ππ* absorption on-set: we attribute this very weak band to direct CT absorption. The extinction coefficient of the potential CT absorption band is very weak ($\varepsilon \cong 10^{-2}$ $M^{-1}cm^{-1}$) and in normal conditions, i.e. **DPTZ-DBTO2** concentrations around 10^{-5} M to 10^{-4} M, this CT absorption is negligible, and the absorption of **DPTZ-DBTO2** is almost entirely given by the sum of the absorptions of the D and A units in **DPTZ-DBTO2**.

In order to assign the new absorption band to a CT transition we have proceeded in the following way:

[S11] H. Tanaka et al, Dual Intramolecular Charge Transfer Fluorescence Derived from a Phenothiazine-Triphenyltriazine Derivative. J. Phys. Chem. C 118, 15985-15994 (2014).

(1) Working at extremely high concentrations, 10^{-2} M in solution, we identified an extra absorption feature in DPTZ-DBTO2 that is not present in the phenothiazine D unit at the same concentrations. The figures 1A and 1B show the absorption spectra of phenothiazine in solvents with increasing polarity, in log scale and linear scale. No significant effect of the solvent polarity is observed in phenothiazine and the onset for the absorption is at 414 nm.

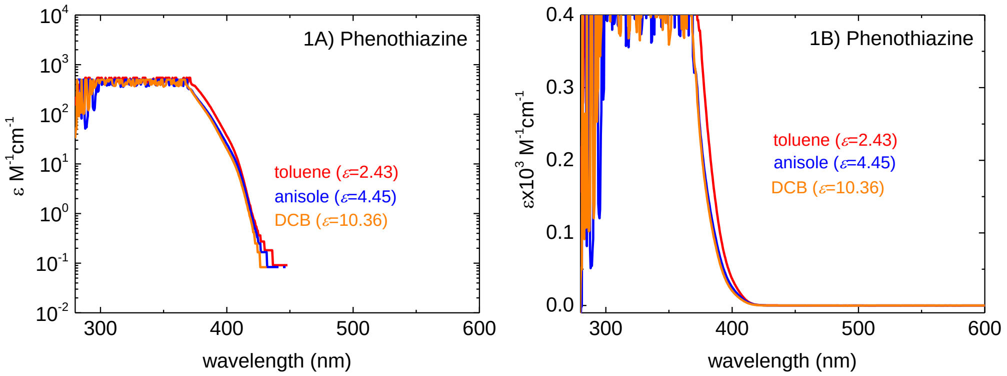

Figure S12-Absoprtion spectra of phenothiazine in three solvents of different polarity.

(2) Collecting the absorption spectra of DPTZ-DBTO2 in toluene solution with increasing concentration, to identify any possible effects of aggregation at such high concentrations. Figure 2A) shows the absorption spectra of **DPTZ-DBTO2** normalized to the absorption at 400 nm. No effect of concentration is observed other than the normal saturation that is visible at higher concentrations and shorter absorption wavelengths. However, the tails of the **DPTZ-DBTO2** absorption spectra excellently match, showing that concentration is not creating new species. Figure 2B) highlights the tail of the **DPTZ-DBTO2** absorption spectra without any normalization, and compares it with the phenothiazine absorption. Clearly the tail of **DPTZ-DBTO2** absorption extends over the phenothiazine D absorption. This is a strong indication for the presence of a CT absorption band.

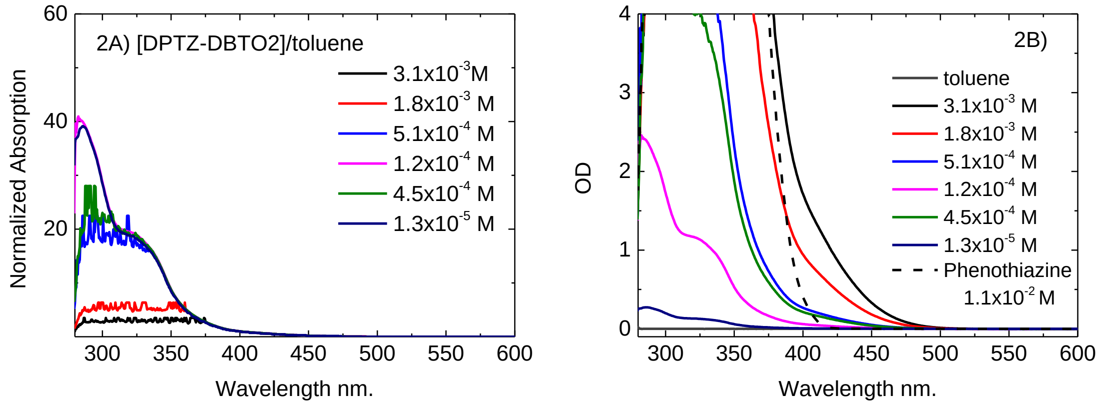

Figure S13-Absoprtion spectra of DPTz-DBTO2 in toluene, obtained at different concentrations.

(3) Determine the extinction coefficient of the CT absorption band in DPTZ-DBTO2, and evaluate the effect of solvent polarity to confirm the CT nature of this transition. Figure 3 shows the absorption of **DPTZ-DBTO2** obtained in solvents with increasing polarity, Fig 3A) in log scale and Fig. 3B) in linear scale. The red-shifted band, relatively to the phenothiazine absorption is clearly observed, and shows a strong blue shift with increasing solvent polarity, clearly indicating that this is an nπ* transition. Given that this new nπ* transition is present only in **DPTZ-DBTO2** and not in the parent (donor fragment) phenothiazine, and also it is extremely weak, concomitant with the near perfect perpendicular orientation of donor and acceptor units in **DPTZ-DBTO2**, we ascribe this new band to CT absorption between the donor and acceptor fragments in **DPTZ-DBTO2**.

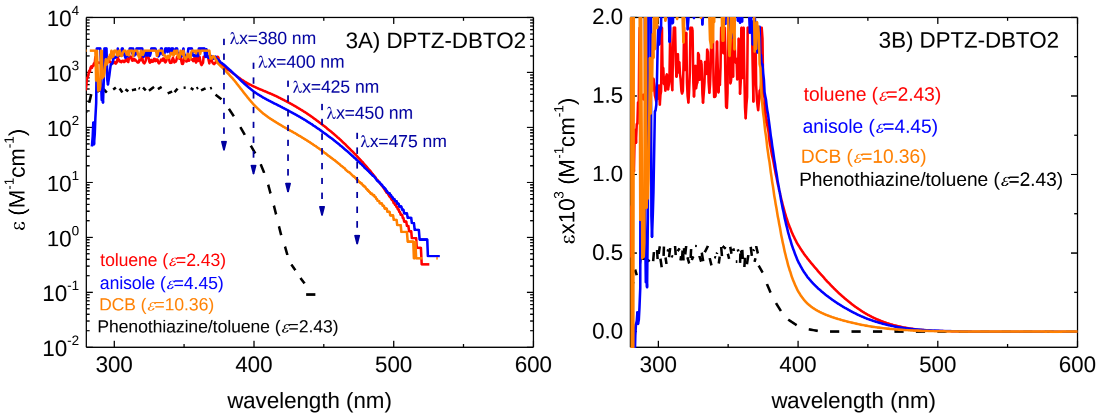

Figure S14- Effect of solvent polarity on the absorption spectra of **DBTZ-DBTO2**.

(4) Selective excitation in the new 'CT absorption band' should give relatively stronger DPTZ-DBTO2 fluorescence quantum yield. The loss mechanism seen when the donor fragment is excited is not accessible when the CT state is directly excited. The PLQY of **DPTZ-DBTO2** (CT band) should therefore increase with excitation at wavelengths longer than the absorption band of the phenothiazine donor fragment. Excitation into the new absorption band clearly leads to emission from the CT state, with an identical spectrum to that seen when the donor is directly excited. Figure 4A), shows that the CT fluorescence intensity increases with increasing excitation wavelength. The emission spectra were obtained simply by changing the excitation wavelength and normalizing for the light absorption at the excitation wavelength (according to equation-S3) on the same solution with front-face (FF) excitation and collection, to avoid the interference of inner-filter effects and reabsorption. Figure 4B), shows the variation of the fluorescence yield with increasing excitation wavelength, relative to the fluorescence yield determined previously with excitation at the phenothiazine D unit (330 nm excitation), i.e. 30%. At longer wavelengths, when the CT state is directly populated the fluorescence yield increases to around 60%. Fig 4B even shows the saturation in the increase of the PLQY as would be expected when moving from mixed donor (ππ*) and CT (nπ*) absorption to pure CT (nπ*) absorption at 475 nm excitation.
As a control experiment, the variation of the fluorescence intensity with increasing excitation wavelength at the absorption edge, was measured also in a solution of phenothiazine at 10^{-2} M. The emission spectra were obtained in exactly the same conditions as for **DPTZ-DBTO2**,

using front-face (FF) excitation and collection, to avoid the interference of inner-filter effects and reabsorption, and in the same range of absorbance. In contrast with **DPTZ-DBTO2**, no increase in the fluorescence intensity was observed in the case of phenothiazine (Figure 4C).

$$I_f = \Phi_f I_a = \Phi_f I_0(1-10^{-OD}) \quad \text{(S3)}$$

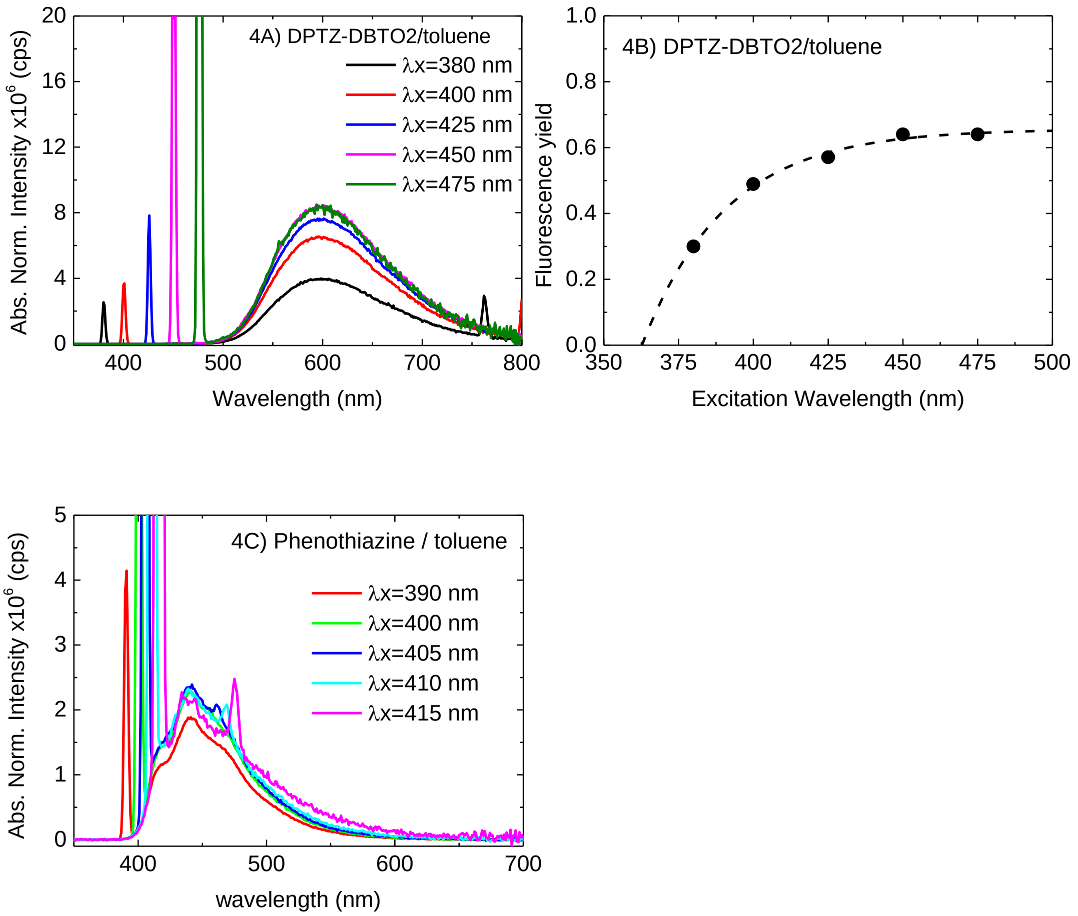

Figure S15-Emission spectra of a) **DPTZ-DBTO2** and c) phenothiazine in toluene with varying excitation wavelength. b) shows the variation of the **DPTZ-DBTO2** fluorescence yield with excitation wavelength.

SI 9. Initial time resolved emission from DPTZ-DBTO2 in solid film

The initial emission from **DPTZ-DBTO2** in CBP host clearly shows ^{1}LE fluorescence characteristic of the donor phenothiazine moiety. This decays with an average lifetime of *ca*. 3 ns and is concomitant with the rise of the 'prompt' ^{1}CT emission. This shows that the electron transfer step is greatly slowed when the D and A fragments are rigidly held perpendicular to one another. Compared to other simple DA molecules (see ref 22) the rate of electron transfer in **DPTZ-DBTO2** is 3 to 4 orders of magnitude slower than in a molecule free to reorganise its geometry.

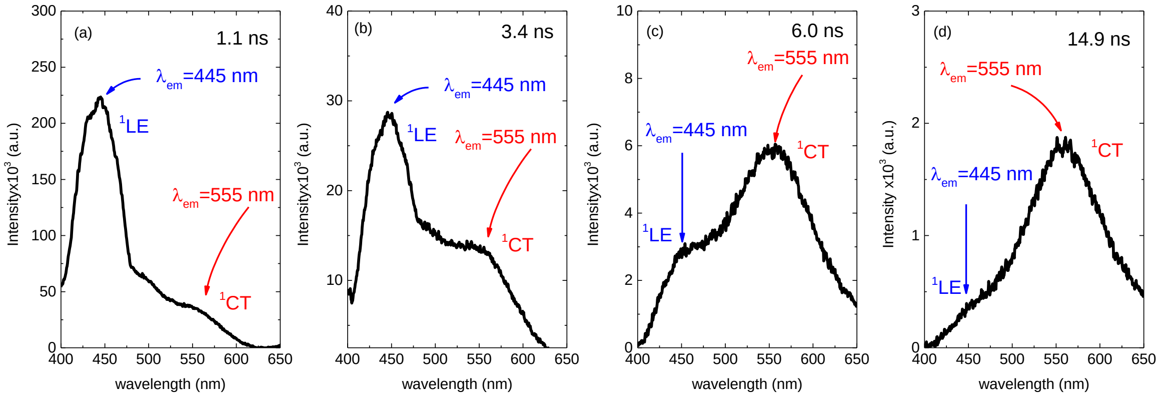

Figure S16-Time resolved emission spectra of **DPTZ-DBTO2** in solid film, obtained at early times following excitation.

SI 10. Intensity dependence of the DPTZ-DBTO2 delayed fluorescence in zeonex solid matrix, and CBP.

The figure shows the intensity dependence of the **DPTZ-DBTO2** delayed fluorescence spectrum (in zeonex) and the emission integral in zeonex and CBP. The fit on the emission integral variation with intensity is obtained with a strictly linear equation (with gradient 1), showing a correlation coefficient of 0.99. Spectra were acquired with 1 μs delay time and integrated for 200 μs.

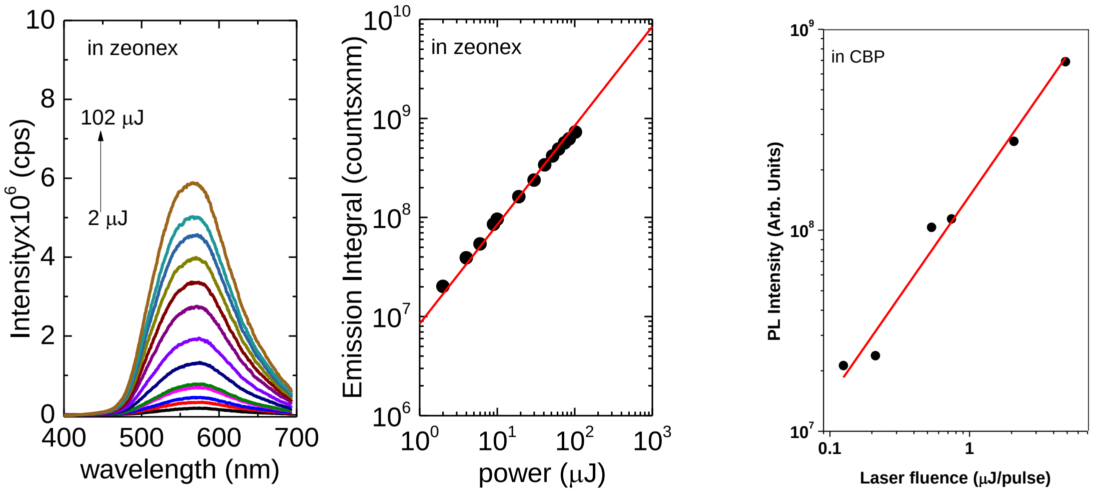

Figure S17-Intensity dependence of the **DPTZ-DBTO2** delayed fluorescence in zeonex solid matrix, and CBP.

SI 11. Temperature dependence of the DF in zeonex matrix.
The temperature dependence is shown of the **DPTZ-DBTO2** delayed emission in zeonex solid matrix, obtained in steady-state conditions. From 320 K to 250 K, the DF intensity appears temperature independent, consistent with a small energy gap between singlet and triplet states. Below 250 K, the DF intensity drops with decreasing temperature, and the energy barrier associated with the reverse intersystem crossing mechanism is determined.

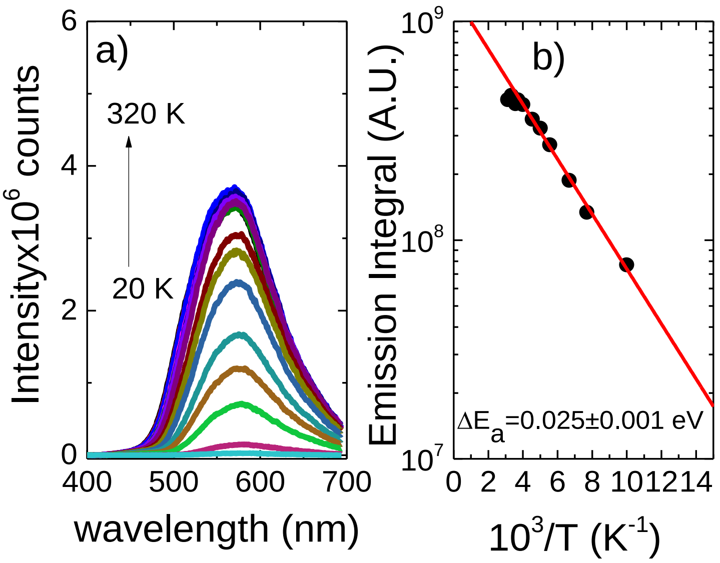

Figure S18-Temperature dependence of the DF in zeonex matrix.

SI 12. Photophysics of DPTZ-DBTO2 in CBP.
Prompt and delayed fluorescence components are clearly separated in time, showing exponential decays. As in solid zeonex matrix, the excited state dynamics of **DPTZ-DBTO2** in CBP are complex, with the emission peak shifting in time, due to underlying contributions from different species. At very early times, less than 3.3 ns, the **DPTZ-DBTO2** fluorescence is clearly dominated by ^{1}LE fluorescence (^{1}D), peaking around 450 nm (2.75 eV), as seen in early time resolved emission spectra, (SI 9), having a decay rate constant of 10^8 s^{-1}. Typically, for D-A ICT systems free to rotate (both planar or twisted) CT formation rates are of order 10^{10}-10^{11} s^{-1} and in some case 10^{12} s^{-1}, 3 to 4 orders of magnitude faster than the rate of 10^8 s^{-1} we measured in **DPTZ-DBTO2**. This effect is again attributed to the rigidity of the D and A units and their near-orthogonally, to achieve very small driving force for electron transfer. The initial D-A electron transfer rate is thus sufficiently slowed for the radiative and non-radiative decay of the ^{1}D fluorescence to compete with the electron transfer process. Following the decay of the initial ^{1}D fluorescence, the **DPTZ-DBTO2** excited state decay proceeds to ^{1}CT prompt fluorescence, which decays completely within the first 100 ns. From 100 ns onwards the **DPTZ-DBTO2** emission is dominated by ^{1}CT delayed fluorescence, which initially peaks at 560 nm, then progressively blue shifts to 512 nm, due again to the underlying ^{3}LE phosphorescence seen at later times.
The temperature dependence of the **DPTZ-DBTO2** emission decay in CBP matrix is given in figure S12. From 298 K to 220 K, the emission decay appears almost independent of temperature, in line with the small energy barrier for triplet harvesting in this material.

However, below 220 K, the ^{1}CT delayed fluorescence is progressively quenched, and at latter times, the long lived ^{3}LE phosphorescence becomes dominant. In all cases, only the delayed ^{1}CT emission shows a temperature dependence indicating that only the delayed ^{1}CT emission arises from TADF.

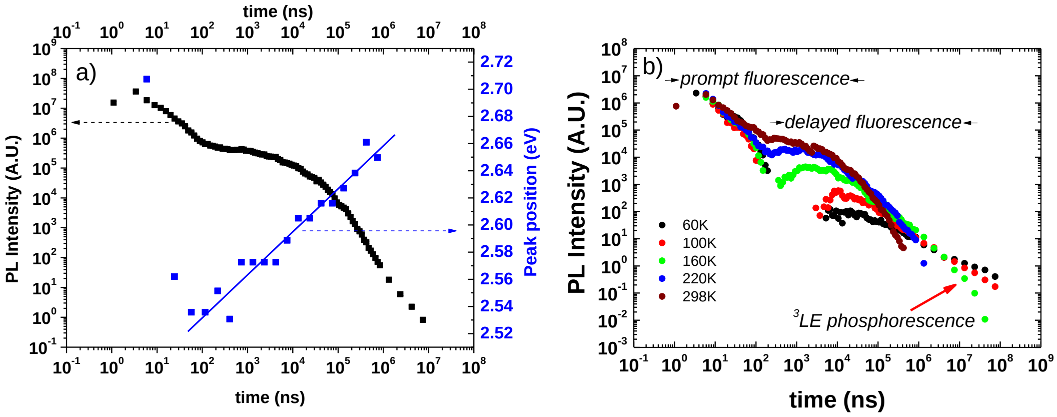

Figure S19-a) Plot of the **DPTZ-DBTO2** emission intensity decay in CBP, over a time interval spanning 9 orders of magnitude, obtained at RT. b) Temperature dependence of the **DPTZ-DBTO2** emission decay in CBP matrix.

SI 13. Electroluminescence spectra obtained from DPTZ-DBTO2:CBP OLEDs.
The figure shows that EL from **DPTZ-DBTO2** devices matches the **DPTZ-DBTO2** CT emission observed in both solution and solid thin film studies.

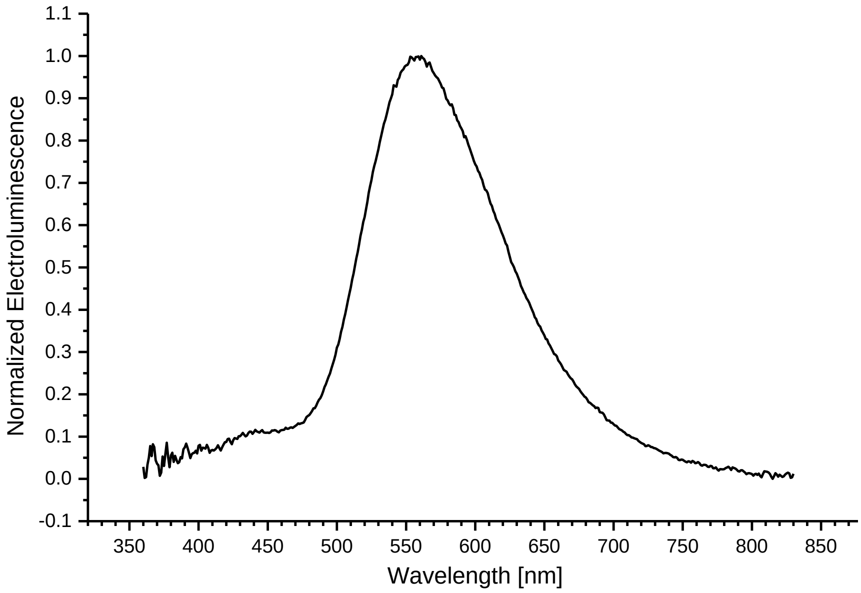

Figure S20-Electroluminescence spectra obtained from **DPTZ-DBTO2**:CBP OLEDs.

SI 14. DPTZ-DBTO2:CBP OLED fabrication.

OLEDs were fabricated with the following structure: ITO/NPB (40 nm)/ 10% **DPTZ-DBTO2**:CBP (30 nm)/TPBi (60 nm)/LiF (1 nm)/Al (70 nm). The OLEDs showed the usual roll-off dependency at the highest observed EQE 18.8% (10 cd/m^2 luminance), and consequently the efficiency decreases significantly above 1000 cd/m^2. However, even with the roll-off effect, the EQE value at 100 cd/m^2 is still very high (16.2%), with 1000 cd/m^2 (11.7%) luminance.

The luminous power efficiency and current efficiency at low luminance are ca. 100 cd/m^2 (21.2 lm/W, 31.8 cd/A, respectively) and high luminance ca.1000 cd/m^2 (18.6 lm/W, 29.5 cd/A).

Figure 8a shows visible trap-controlled, space-charge limited conduction behaviour with a steep rise of current above 2.9 V. The current turn-on voltage is slightly lower than the luminance turn-on voltage of 3.1 V (Fig. 8a). This difference is probably due to the small intensity of emitted light at low current.

The maximum brightness for the **DPTZ-DBTO2** device is 31060 cd/m^2 at 11.8 V (Fig.8a), showing good charge carrier balance. The very low drive voltage of this device leads to a luminous power efficiency of >20 lm/W (Fig. 8b), corresponding to applicability requirements of OLEDs. Initially, the device exhibits a high (34 cd/A) level of current efficiency, which starts to decrease above 3000 cd/m^2 (Fig. 8c).

The energy-level diagrams in Figure 8d define some of the electronic structure parameters relevant to designing organic materials for OLEDs. CBP was chosen as the host for **DPTZ-DBTO2**, because of promising preliminary photophysical analysis; the NPB layer was used as a hole injecting and transporting layer due to the fact that it has a similar HOMO value to CBP yielding good hole transport. TPBi was chosen as the electron transporting layer because of its high electron and low hole mobilities, although it is not a good hole blocking layer in this application. Employing a separate HBL, such as BCP or OXD-7, did not improve the EQE (similar 18% values were obtained), but the power efficacy is reduced when OXD-7 or BCP layers are used. From the energy diagram it is clear that both holes and electrons are directly injected into the CBP layer and then populate the **DPTZ-DBTO2** layer. Layer thicknesses were chosen experimentally and were not further optimised.

Luminance (cd m^{-2})	Voltage (V)	EQE (%)	Luminous power efficiency (lm W^{-1})	Current efficiency (cd A^{-1})
**100**	4.2	16.2	21.2	31.8
**1000**	5.0	11.7	18.6	29.5

SI 15. Synthesis of DPTZ-DBTO2

2,8-Dibromodibenzothiophene-*S*,*S*-dioxide (500 mg, 1.345 mmol), phenothiazine (563 mg, 2.824 mmol), $Pd_2(dba)_3$ (80 mg, 0.087 mmol) and XPhos (83 mg, 0.175 mmol) were dissolved in dry toluene (20 mL) and degassed for 30 min, then ^{t}BuONa (284 mg, 2.959 mmol) was added and the reaction mixture degassed for a further 15 min. The reaction was stirred overnight at 110 °C. Upon cooling, the mixture was diluted with toluene (50 mL) and

washed with water, dried over Na_2SO_4, filtered and the solvent was removed at low pressure. The resulting residue was chromatographed (silica gel, eluent hexane/DCM 1:2 v/v) to give compound **DPTZ-DBTO2** as a yellow solid (68%). ^{1}H-NMR (400 MHz, DMSO-d$_6$): δ = 7.99 (d, J_1 = 8.4 Hz, 2H), 7.69 (d, J_2 = 2.1 Hz, 2H), 7.39 (dd, J_3 = 7.7, J_4 = 1.5 Hz, 4H), 7.33 (dd, J_1 = 8.4, J_2 = 2.1 Hz, 2H), 7.28-7.21 (m, 4H), 7.16 (td, J_3 = 7.7, J_6 = 1.3 Hz, 4H), 7.01 (dd, J_5 = 8.1, J_6 =1.3 Hz, 4H). ^{13}C-NMR (100 MHz, DMSO-d$_6$): δ = 148.4, 141.3, 132.9, 132.7, 128.0, 127.8, 127.4, 125.3, 123.9, 123.8, 122.6, 114.9. MS-ASAP$^+$: *m/z* (%)= 611.1 ([M+H]$^+$, 100%). HRMS-ASAP$^+$ *m/z* calculated for $C_{36}H_{23}N_2O_2S_3$: [M+H]$^+$ 611.0922, found: 611.0915. Mpt. decomposes > 350 °C.

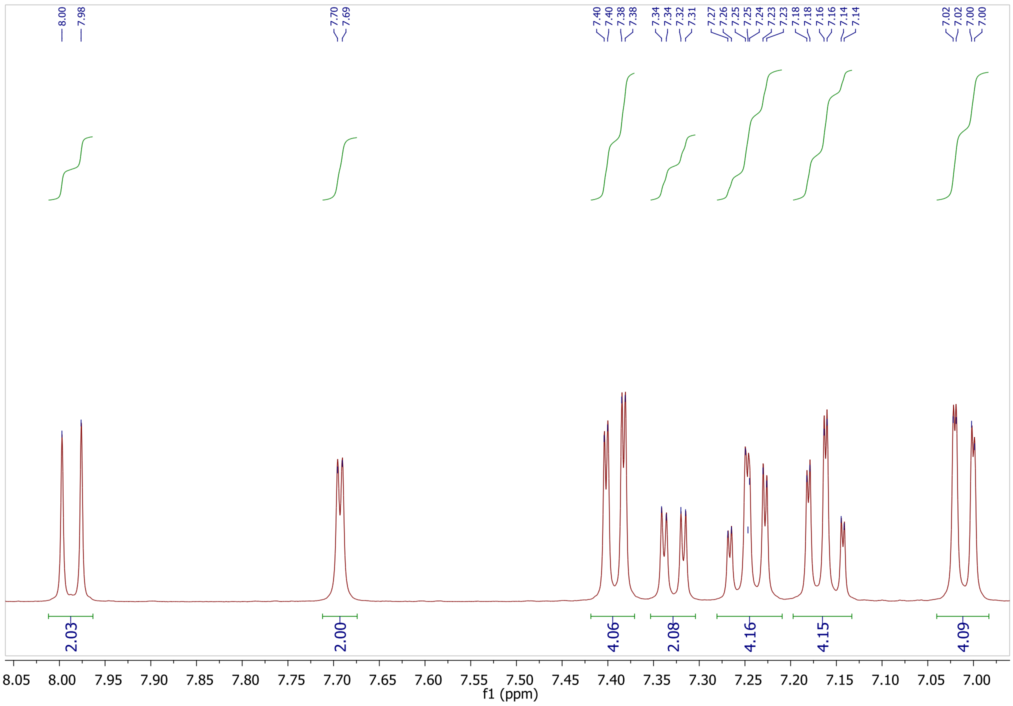

Figure S21-^{1}H NMR spectrum of **DPTZ-DBTO2** in DMSO-d$_6$

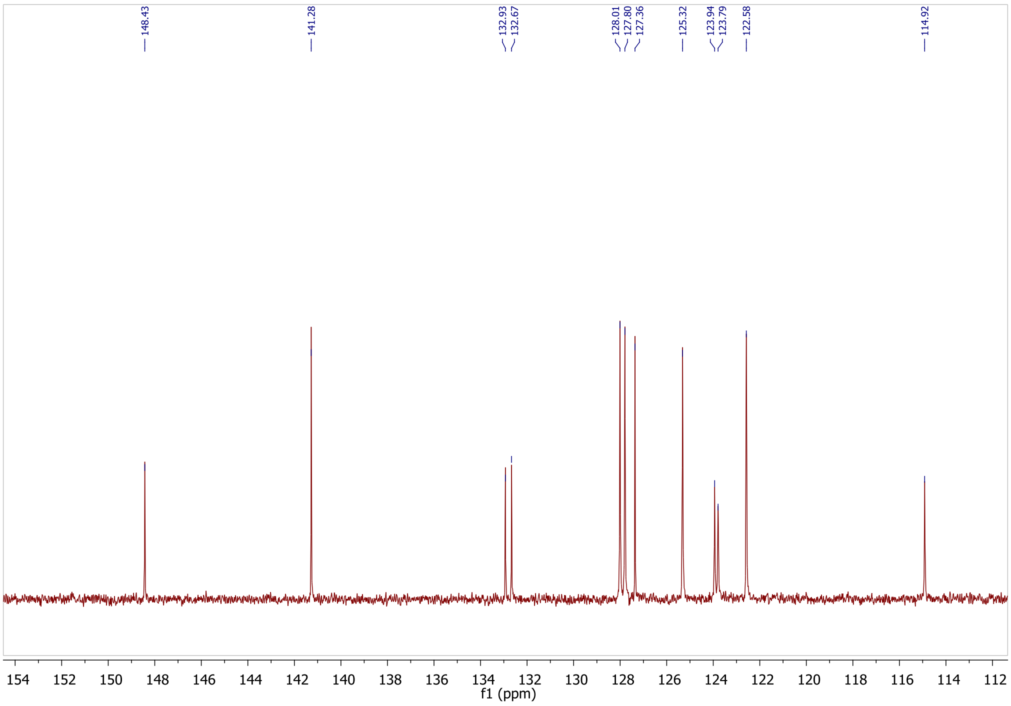

Figure S22-^{13}C NMR spectrum of **DPTZ-DBTO2** in DMSO-d_6

SI 16. Additional X-ray crystallographic data.

The molecular structure of **DPTZ-DBTO2**$\cdot$2$CDCl_3$ reported herein is similar to the non-solvated structure reported by Gan et al[S12] during preparation of this manuscript.

[S12] Gan, S., Luo, W., He, B., Chen, L., Nie, H., Hu, R., Qin, A., Zhao, Z., Tang, B. Z., Integration of aggregation-induced emission and delayed fluorescence into electronic donor-acceptor conjugates. J. Mater. Chem. C. **4**, 3705-3708 (2016).

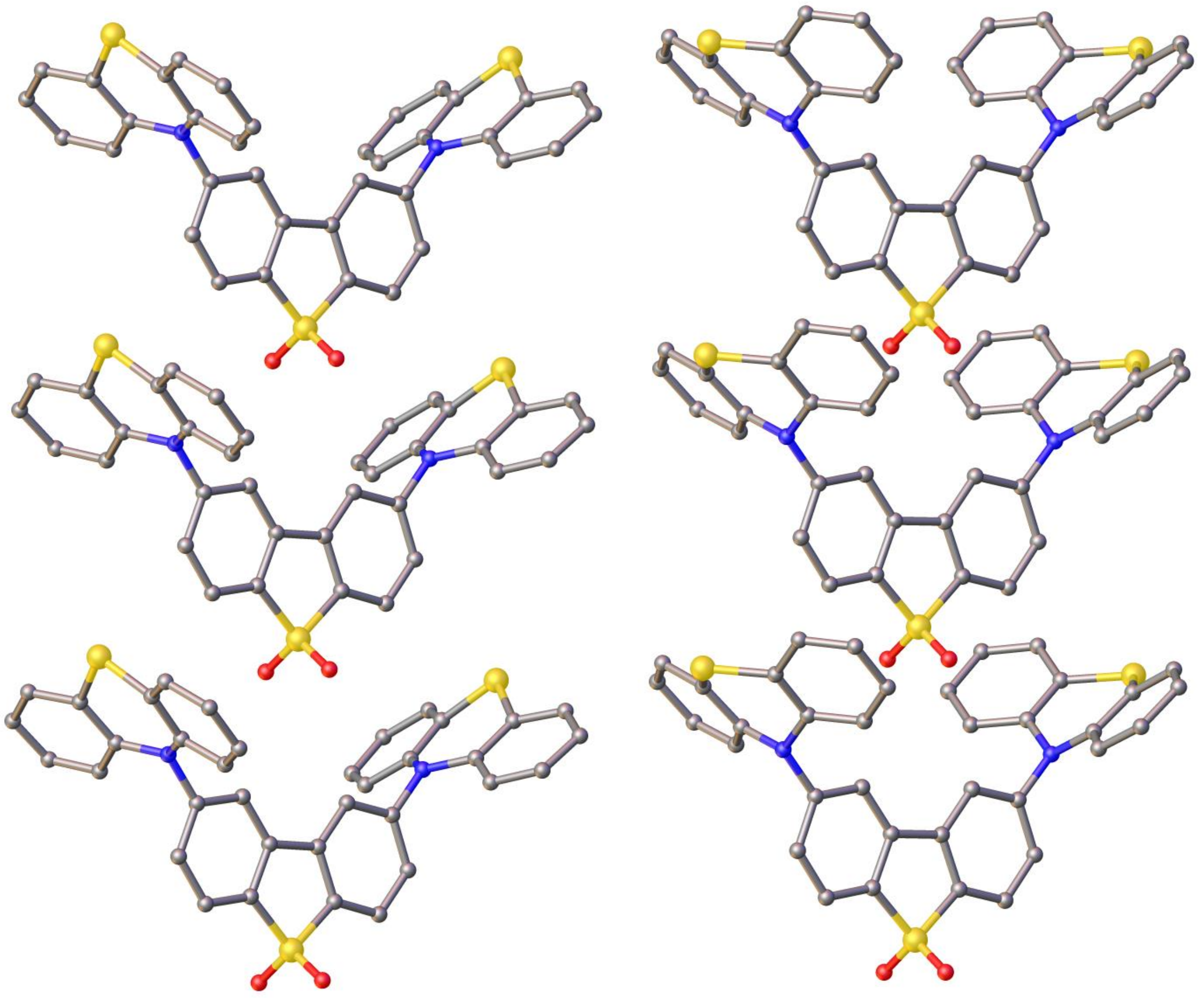

Figure S23-Additional X-ray crystallographic data of **DPTZ-DBTO2**.

Columns of molecules striding a twofold axis, are the main motifs in the crystal structures of **DPTZ-DBTO2** (left)[S12] and **DPTZ-DBTO2**·2$CDCl_3$ (right). The periodicity along the chain (= lattice parameter *b*) is practically the same, 7.43 and 7.42 Å, respectively. Note that the phenothiazine moieties are folded outward in **DPTZ-DBTO2** but inward in **DPTZ-DBTO2**·2$CDCl_3$.
